# Enrichment Probe Sets Combining Universal and Lineage‐Specific Targets Help Resolve Recalcitrant Lineages

**DOI:** 10.1111/1755-0998.70173

**Published:** 2026-07-28

**Authors:** Irene Villa‐Machío, Irene Masa‐Iranzo, Nicolai M. Nürk, Lisa Pokorny, Andrea S. Meseguer

**Affiliations:** ^1^ Real Jardín Botánico (RJB‐CSIC) Madrid Spain; ^2^ Plant Systematics, Bayreuth Centre of Ecology and Environmental Research (BayCEER) University of Bayreuth Bayreuth Germany

**Keywords:** clusioid clade, Hyb‐Seq, Malpighiales, phylogenomics, probe design

## Abstract

The combination of target capture sequencing (TCS) with low‐coverage whole genome sequencing (lcWGS), an approach known as Hyb‐Seq, has allowed the integration of natural history collections into the genomics revolution, transforming biodiversity research. To implement Hyb‐Seq, a collection of genomic targets is needed to design probes. In flowering plants, the Angiosperms353 kit has been proven effective at multiple evolutionary scales, with limitations. Malpighiales is one of the most challenging flowering plant orders to resolve. Within this order, the clusioid clade (~2.200 species, 94 genera, five families) is no exception. To resolve phylogenetic relationships in this recalcitrant clade, we design a custom probe set composed of 39,936 120‐mer probes targeting 626 nuclear orthologs. The Clusioids626 kit includes all Angiosperms353 targets and 273 clusioid‐specific ones, carefully chosen taking copy‐number, length evenness, and phylogenetic informativeness into account. We tested our probe set on 70 accessions representing all clusioid families and tribes. On average, 50.4% reads mapped to our targets, recovering a median of ~600 orthologs/sample. Relationships for all clusioid families are fully resolved for our nuclear targets. A Hypericaceae‐Podostemaceae clade is sister to Calophyllaceae, which are all sister to Bonnetiaceae, and then to Clusiaceae. Additionally, we retrieved 105 plastid coding sequences from the lcWGS fraction with a custom target file, evidencing strong incongruence between nuclear and plastid topologies. The Clusioids626 kit performs better than the Angiosperms353 one alone. Our design workflow can be extended to other lineages for which a universal probe set exists but more resolution is needed.

## 1 Introduction

Target capture sequencing (TCS) has emerged as a standard phylogenomic approach in systematics and evolution (McKain et al. 2018; Andermann et al. 2020). In particular, TCS combined with low‐coverage whole genome sequencing (lcWGS), an approach known as Hyb‐Seq (Weitemier et al. 2014; Dodsworth et al. 2019), can result in hundreds to thousands of nuclear genomic loci (e.g., nuclear orthologs), as well as high‐copy number DNA (e.g., organellar DNA), useful for phylogenomic inference in non‐model organisms (Breinholt et al. 2021; Ametrano et al. 2025). This technique has recently become very popular, as it has been shown to be both cost efficient (Hale et al. 2020) and effective in resolving phylogenetic relationships for flowering plants across evolutionary scales (from micro to macroevolutionary levels; Villaverde et al. 2018; Zuntini et al. 2024) and for DNA templates of varying quality, including degraded herbarium material (Brewer et al. 2019; Shee et al. 2020). The wealth of molecular data generated by this technique has been proven useful for generating robust phylogenetic hypotheses and to resolve species radiations (Kadlec et al. 2017; Mitchell et al. 2017; Larridon et al. 2020), but also to investigate gene tree conflict and the causes leading to reticulation, whether methodological (e.g., gene tree estimation error) or biological, i.e., incomplete lineage sorting (ILS), horizontal gene transfer, hybridization, etc. (Fleming et al. 2023; Steenwyk et al. 2023; Bjornson et al. 2024).

Gene capture techniques have followed two main strategies. One approach relies on universal hybridization capture kits designed to target large clades across the tree of life (Buddenhagen et al. 2016; Breinholt et al. 2021; Waycott et al. 2021). The most popular in plants being the Angiosperms353 kit designed to target all flowering plants (Johnson et al. 2019). This universal kit resolves deep phylogenetic relationships across distantly related taxa, and has also demonstrated utility in resolving shallow relationships (Larridon et al. 2020; Slimp et al. 2021; Beck et al. 2021; Balant et al. 2025), though their effectiveness at finer scales remains debated for certain lineages (Yardeni et al. 2022). This has led to the development of customized probe sets tailored to specific plant lineages. To date, custom kits are available for numerous plant families, such as Euphorbiaceae (Villaverde et al. 2018), Dioscoreaceae (Soto Gomez et al. 2019), Ochnaceae (Schneider et al. 2021), Scrophulariaceae (Villaverde et al. 2023) or Asteraceae (Moore‐Pollard et al. 2024), among others. More recently, efforts to combine universal and lineage‐specific probe sets into a single kit have shown great promise (Eserman et al. 2021; Ogutcen et al. 2021; Fonseca et al. 2023, 2024; Bentz and Leebens‐Mack 2024; Musker et al. 2025). These probe sets integrate the broad applicability of universal kits with the high resolution of specific ones, providing an efficient and versatile framework for phylogenomic studies (Fonseca et al. 2024). However, the use of kits combining universal and specific probe sets is still quite limited, in spite of their potential.

The order Malpighiales accounts for ~7.80% of eudicot diversity and includes over 16,000 species, across ~700 genera and 36 families (APG IV 2016; Zuntini et al. 2024). This order has been referred to as one of the most recalcitrant angiosperm groups, since resolving phylogenetic relationships at various levels has proven highly challenging (Wurdack and Davis 2009). Phylogenetic relationships in this group remain poorly understood, even with inferences based on ~100 genes from three genomic compartments (Xi et al. 2012). This difficulty has been attributed to its rapid radiation in the Mid‐Cretaceous (Davis et al. 2005; Xi et al. 2012) and the challenges of phylogenetic inference, referred to as a ‘perfect storm’ (Cai et al. 2021) of ILS, introgression, and gene tree estimation error that blurs the resolution of Malpighiales, particularly with regards to the backbone topology. Within order Malpighiales, the clusioid clade comprises ~2.2 thousand species, 94 genera, and five families: Bonnetiaceae, Calophyllaceae, Clusiaceae, Hypericaceae, and Podostemaceae (Wurdack and Davis 2009; Ruhfel et al. 2011; Xi et al. 2012). The clusioid clade is nearly cosmopolitan, although the majority of its species are found in the tropics, in a broad range of habitats—closed canopy rainforests, dry, temperate, and high‐altitude tropical forests, and swift‐flowing aquatic environments—and forms, e.g., trees, shrubs, and herbs (Stevens 2007; Ruhfel et al. 2016; Meseguer et al. 2018). The terrestrial members of the clade are key components of tropical rainforest ecosystems (Davis et al. 2005); while Podostemaceae, as the largest strictly aquatic plant family of the clade, plays an essential role in riverine systems (Philbrick and Novelo 1995; Cook 1996). Several species of the clusioid clade are economically important, including edible species (e.g., the mangosteen, *Garcinia mangostana* L., and the mammee apple, *Mammea americana* L.) or others used for timber production ( *Calophyllum brasiliense* Cambess., *Mesua ferrea* L.), pharmaceutical purposes ( *Hypericum perforatum* L.), or in horticulture (several Calophyllaceae, Clusiaceae, and Hypericaceae species) (Ernst 2003; Stevens 2007; Ruhfel et al. 2011).

Relationships within the clusioid clade, based on morphological data, were so unclear that the existence of the group itself was contested. Families Calophyllaceae and Hypericaceae were nested within Clusiaceae *s.l*., also known as Guttiferae (e.g., Robson 1977; Notis 2004), and some Podostemaceae genera were placed in distantly related orders, such as Theaceae (Ericales, Wawra 1886; Cronquist 1981; Takhtajan 1997), making the placement of Podostemaceae uncertain and speculative. In the last decades, several molecular studies have sought to disentangle relationships of Malpighiales and the clusioid clade (Korotkova et al. 2009; Wurdack and Davis 2009; Ruhfel et al. 2011; Jin et al. 2020; Cai et al. 2021) and within clusioid families (Calophyllaceae: Trad et al. 2021; Clusiaceae: Gustafsson et al. 2002; Marinho et al. 2019; Gaudeul et al. 2024; Hypericaceae: Meseguer et al. 2013, 2015; Nürk et al. 2013, 2015; Podostemaceae: Tippery et al. 2011; Bedoya et al. 2019). However, despite the advances recently made, reconstructing phylogenetic relationships within the clusioid clade remains challenging, with the topology changing depending on the molecular markers used or the analytical approach implemented.

Wurdack and Davis (2009), relying on 13 gene regions from all three plant genomic compartments, confirmed the monophyly of the clusioids and recovered a Bonnetiaceae plus Clusiaceae clade sister to a clade composed of Calophyllaceae sister to Hypericaceae plus Podostemaceae. Ruhfel et al. (2011) obtained the same topology using three plastid and one mitochondrial genes, with the most extensive sampling to date (194 spp.). So did Xi et al. (2012) using genes from all three genomic compartments, and Jin et al. (2020) using 82 protein‐coding plastid genes. Based on plastomes, Trad et al. (2021) mostly supported the topology of Wurdack and Davis (2009), but highlighted major gene tree discordance, e.g., at the divergence between Bonnetiaceae and Clusiaceae. Recently, Cai et al. (2021) presented a different topology based on 423 nuclear genes, and showed extensive gene tree discordance. This latter topology did not include Podostemaceae and showed Clusiaceae successively sister to Hypericaceae, and a Bonnetiaceae plus Calophyllaceae clade. In general, relationships within the Bonnetiaceae, Clusiaceae, and Podostemaceae families are largely consistent across different studies, but this is not the case for all other clusioid families (Ruhfel et al. 2011; Trad et al. 2021).

In this study, we design a new custom probe set, the Clusioids626 kit, which combines nuclear ortholog genes specific to the clusioid clade with genes from the universal Angiosperms353 enrichment panel. Additionally, we develop a specific plastid target file for the clusioid clade to recover plastid genes from Hyb‐Seq off‐target reads. The Clusioids626 kit, together with the plastid target file, was employed to infer phylogenetic relationships within the clusioid clade. We compare the performance of our Clusioids626 probe set against that of the Angiosperms353 universal panel. Finally, we also analyse gene tree conflict within and across genomic compartments.

## 2 Materials and Methods

### 2.1 Taxon Sampling for Custom Probe Set Design

To design the Clusioids626 probe set we used 12 transcriptomes, one belonging to Ochnaceae (putatively sister to the clusioid clade; Zuntini et al. 2024) and 11 belonging to the five clusioid families: five Hypericaceae, three Calophyllaceae, two Clusiaceae, and one Bonnetiaceae (Table S1). Transcriptomic data were downloaded from the sequence read archive (SRA) database, hosted by the National Center for Biotechnology Information (NCBI), and were assembled de novo using Trinity v.2.9.1 (Grabherr et al. 2011) from quality‐filtered reads obtained using Trimmomatic v.0.36 (Bolger et al. 2014). Unless otherwise stated, all software was run with default settings.

### 2.2 Probe Set Design and Loci Discovery

Two pipelines were utilized to select and mine target loci: MarkerMiner v.1.0 (Chamala et al. 2015) and HybPiper v.2.1.6 (Johnson et al. 2016). First, we identified putative single‐copy nuclear orthologs using MarkerMiner. This pipeline relies on the curated list of 2809 mostly single‐copy genes (De Smet et al. 2013) from the PLAZA Comparative Genomics Platform (Van Bel et al. 2012). As genomic inputs, we used the 12 transcriptome assemblies listed above and *Ricinus communis* L. (Euphorbiaceae, Malpighiales; v.0.1; Chan et al. 2010) as reference genome, available from Phytozome (Goodstein et al. 2012). As a result, a total of 1637 nuclear orthologs shared across clusioids were identified (Table S2). Subsequently, we examined whether our list of candidate gene sequences overlapped with those included in the universal Angiosperms353 enrichment panel (Johnson et al. 2019) using BLAST+ (Camacho et al. 2009). Out of the 353 single‐copy targets of the Angiosperms353 enrichment panel, 217 were not recovered by MarkerMiner. Second, we used the HybPiper pipeline, which requires a reference (i.e., a fasta file with the intended targets), to mine the remaining 217 targets, which we got from the mega353.fasta target file (McLay et al. 2021; available from github.com/chrisjackson‐pellicle/NewTargets). We generated an ad hoc clusioid target file that included all the gene sequences extracted from MarkerMiner, plus the gene sequences from the mega353.fasta target file. The 12 transcriptomes were then mined with this ad hoc clusioid target file to obtain matrices with sequence information for each of the selected target genes. These target‐gene data matrices were aligned with MAFFT v.7.520 (Katoh and Standley 2013), and summary statistics were calculated with AMAS v.1.0 (Borowiec 2016). For probe design, final loci were selected that met the following criteria: (i) all loci contained sequences of at least seven species; (ii) three of them belonged to *Hypericum*, the most species‐rich genus in the clusioid clade (WFO 2024); (iii) minimum target length of 350 bp; and (iv) at least 0.08 phylogenetically informative sites per target gene. These filters resulted in a final set of 626 targets.

Biotinylated RNA probes were designed by Daicel Arbor Biosciences (Ann Arbor, MI, USA). Malpighiales Phytozome genomes *Linum usitatissimum* L. v.1.0, *Manihot esculenta* Crantz v.8.1, *Populus trichocarpa* Torr. & A.Grey ex Hook. v.4.1, *R. communis* v.0.1, and *Salix purpurea* L. v.5.1, together with NCBI genomes *Bruguiera* Lam., *Hevea* Aubl., *Hypericum*, and *Viola* L., were used to remap the probes and to check that they did not overlap with high‐copy‐number elements, organellar DNA inclusive.

The final set had 39.9 thousand 120‐mer probes with 1× tiling density. Given this tiling density and to ascertain the efficacy of the probes in recovering our clusioid targets, BLAST+ was run on the probes against said target sequences. Various parameters were calculated, including the percent identity (i.e., percentage of nucleotides that are the same between the two sequences), alignment length, and percent coverage. The mean percent coverage of the probes for each target was extracted using the command‐line program GNU datamash (FSF 2014).

### 2.3 Taxon Sampling, Genomic Library Preparation, and Probe Set Validation

To validate our probe set, we used 70 samples, 67 belonging to seven families of Malpighiales (Table S3) and three to order Celastrales. Out of those 70 samples, 38 were processed in‐house with the Clusioids626 kit (see molecular workflow below) and 32 were downloaded from the European Nucleotide Archive (ENA) and mined for our 626 targets (see bioinformatic pipeline below). The clusioid clade was represented by 58 species from its five families: 19 Calophyllaceae, 19 Hypericaceae, seven Podostemaceae, 10 Clusiaceae, and three Bonnetiaceae (Table S3). Species from other putatively closely related Malpighiales families were included as outgroups (six Ochnaceae, three Linaceae; Table S3). Species names follow WFO (2024).

Genomic DNA was extracted from 38 herbarium collections (Table S3) following the CTAB protocol (Doyle and Doyle 1987) with modifications (Larridon et al. 2020; Shee et al. 2020). All DNA extracts were quantified using a Qubit 3 Fluorometer with dsDNA high sensitivity (HS) assay kits (ThermoFisher Scientific, Waltham, MA, USA). Genomic DNA with fragment sizes ≥ 500 bp (prior to sonication) was sheared to an average fragment size of ~250 bp using a Covaris M220 Focused‐ultrasonicator (Woburn, MA, USA).

Libraries were prepared using the DNA NEBNext Ultra II Library Prep Kit and Dual Index Primers (Multiplex Oligos) Sets 1 and 2 for Illumina (New England BioLabs, Ipswich, MA, USA), following the manufacturer's protocol, but adjusting for half volume reactions (Hale et al. 2020). After end‐repair, adapter ligation, and cleaning, libraries were indexed (10‐cycle polymerase chain reaction, PCR), and then cleaned with AMPure XP magnetic beads (Beckman Coulter Life Sciences, Indianapolis, IN, USA). The samples were then quality‐checked with a Bioanalyzer High Sensitivity DNA chip on a 2100 Bioanalyzer (Agilent Technologies, Santa Clara, CA, USA) and quantified using the Qubit as above. Genomic libraries were multiplexed in equimolar pools for target capture.

Pools were enriched using the Clusioids626 kit following the manufacturer's myBaits Manual v.5.02. Hybridizations were performed at 65°C for all genomic libraries. The enriched pools were amplified for 12, 14, or 16 PCR cycles (depending on DNA template quality) using the primers P5 and P7 for Illumina (Meyer and Kircher 2010), and PCR products were cleaned with AMPure XP beads. Final products were again run on the Bioanalyzer as above, and then they were pooled equimolarly for sequencing. After multiplexing 75% enriched library pools with 25% plain genomic libraries, for all species, sequencing was performed on an Illumina HiSeq (San Diego, CA, USA) at Macrogen (Seoul, South Korea), producing 2 × 150 bp long reads.

### 2.4 Quality Filtering of Raw Reads and Sequence Assembly

Demultiplexed reads were inspected before and after trimming with FastQC 0.12.1 (Andrews 2010) for quality control. Raw reads were paired, adapter cleaned, and trimmed using fastp v.0.23.4 (Chen et al. 2018) with settings: −f 15, −t 10, ‐F 15, −T 15, −g, ‐W 4, −r, −M 20, −q 15, −l 50 (see https://github.com/OpenGene/fastp/blob/master/README.md for details). Sequences were recovered using HybPiper with our Clusioids626 target file as the reference. The trimmed and paired reads were initially mapped against the target loci using BWA v.0.7.17 (Li and Durbin 2009), followed by de novo assembly using SPAdes v.3.15.5 (Bankevich et al. 2012). HybPiper assembly was repeated for samples that recovered less than 100 genes with sequences > 50% of the mean target length, lowering the coverage cutoff down to 3. When gene recovery was low, samples were rerun using BLASTx (Camacho et al. 2009) instead of BWA. The presence of potential paralogs was investigated using the paralog_retriever parameter and potential paralogs were removed if they had > 2 sequences/gene in ≥ 2 samples.

### 2.5 Data Matrix Construction and Multiple Sequence Alignment

We retrieved FASTA files (HybPiper retrieve_sequences script) containing the exon‐only sequences for 70 samples to build the corresponding nuclear data matrices. To identify and discard suboptimal samples and/or regions, and to build balanced data matrices (Shee et al. 2020), we implemented in R v.4.2.1 (R Core Team 2024) the max_overlap script (available from github.com/keblat/bioinfo‐utils/blob/master/docs/advice/scripts/max_overlap.R). We filtered out underrepresented, incomplete, and unevenly distributed sequences and species (coverage score < ⅔ median value) to reduce the noise in our matrices.

Sequence matrices were aligned in MAFFT. We computed the summary statistics of the individual MSAs with AMAS and removed genes with values < ⅓ of the median for alignment size and proportion of Parsimony‐informative characters (*P*_PIC_). Exploratory gene trees were generated using FastTree v.2.1.11 (Price et al. 2010) and analysed in TreeShrink v.1.3.7 (Mai and Mirarab 2018) with the ‘per‐species’ option, to automatically remove outlier taxa by excluding branches that increased the diameter of each gene tree beyond a given threshold (α parameter). Summary statistics were calculated with AMAS for all data matrices, so that we could choose the *α* parameter (0.50 threshold) that resulted in the highest proportion of *P*_PIC_, the lowest proportion of missing data, and the smallest data matrix size. Each locus was then realigned using MAFFT. We further refined the MSAs by trimming each gene using trimAl v.1.4.1 (Capella‐Gutiérrez et al. 2009) with relaxed settings (−gt 0.3 ‐cons 30) and, once more, summarized the MSAs stats with AMAS. We removed genes whose alignment size and *P*_PIC_ median values were < ⅓.

### 2.6 Phylogenomic Analyses

To reconstruct phylogenetic relationships in the clusioid clade, we implemented both multispecies coalescent (MSC) and maximum likelihood estimation (MLE) approaches.

#### 2.6.1 Nuclear Tree Inference

Shrunk and trimmed MSAs were employed to generate MLE gene trees using IQ‐TREE2 v.2.2.3 (Minh et al. 2020). The best substitution model for each gene was selected according to ModelFinder Plus (MFP; Kalyaanamoorthy et al. 2017). The resulting collection of MLE unrooted gene trees was used to infer the species tree with ASTRAL‐III v.5.7.8 (Zhang et al. 2018) under the MSC approach. Before running ASTRAL‐III, branches in gene trees with low support were collapsed using the nw_ed function from the Newick Utilities v.1.6 toolkit (Junier and Zdobnov 2010) to improve the accuracy of species tree inference (Mirarab 2019; Simmons and Gatesy 2021). Branch support in ASTRAL‐III was calculated as local posterior probabilities (LPPs), and gene congruence was evaluated using normalized quartet scores (QS) values. These scores, which range from 0 to 1, represent the proportion of input gene tree quartets that are consistent with the species tree (the higher the score, the less discordant the gene trees are). We implemented the *astralProjection* function, available from the *AstralPlane* package (Hutter 2021) in R to plot pie charts depicting these QS values. An additional nuclear MLE analysis was conducted in IQ‐TREE2 by concatenating all shrunk and trimmed MSAs into a supermatrix with AMAS. Genes were partitioned and allowed to evolve at independent rates and under different substitution models (determined by MFP). Support for the concatenated nuclear MLE tree was calculated (1000 replicates) using ultrafast bootstrap (UFB; Hoang et al. 2018) and SH‐like approximate likelihood ratio tests (SH‐aLRT; Anisimova et al. 2011). All phylogenetic trees were visualized and plotted with FigTree v.1.4.4 (Rambaut 2018).

#### 2.6.2 Amino Acid Plastid Target File and Organellar Tree Inference

We mined plastid coding DNA sequences (CDS) from quality‐checked paired reads of 70 clusioid clade samples and related taxa using HybPiper and a custom‐designed AA plastid target file comprising 105 CDS. The stepwise design of this AA plastid target file started with the complete plastome sequences (downloaded from NCBI) of 14 species belonging to all clusioid families (Table S4). Following a first‐pass mining of our 70 samples in HybPiper, we added the sequences of 13 more samples into our final AA plastid target file. These 13 samples were selected to reduce phylogenetic biases; that is, samples were chosen to maximize phylogenetic spread. The recovered CDS were concatenated into a single data matrix and a MLE plastid tree was inferred using IQ‐TREE2, partitioned by CDS, and MFP was used to select the best substitution model for each CDS. Support was calculated using UFB and SH‐aLRT (1000 replicates).

### 2.7 Probe Sets Comparison: Clusioids626 vs. Angiosperms353

To evaluate the performance of our custom Clusioids626 probe set against the universal Angiosperms353 enrichment panel, we compared HybPiper summary statistics, as well as phylogenetic support and resolution of nuclear phylogenies inferred for data matrices mined with both our nuclear Clusioids626 and mega353.fasta target files for all 70 samples. Nuclear matrices containing exon‐only sequences were shrunk and filtered following the same procedure described above.

### 2.8 Assessing Gene‐ Vs. Species‐Tree and Cyto‐Nuclear Conflict

We assessed topological discordance and conflict between the MSC nuclear and MLE plastid ultrametric topologies (obtained in R with the *chronos* function from the *ape* package; Paradis and Schliep 2019) using the *tanglegram* and *untangle* functions (*step1side* method) from the *dendextend* package (Galili 2015) in R.

For the nuclear dataset, we also performed network analyses to explore potential gene trees vs. species tree conflicts and to detect patterns of reticulation caused by hybridization and/or ILS. The concatenated‐genes matrix, with 552 loci and a selection of 58 samples (including only representatives of the five clusioid families), was imported into SplitsTree v.6.3.35 (Huson and Bryant 2024) using the neighbour‐net algorithm with uncorrected P‐distances to compute a split network (1000 bootstrap replicates). To further explore the contribution of hybridization in the evolutionary history of the clusioid clade, we inferred phylogenetic networks using PhyloNet v.3.8.2 (Wen et al. 2018), which enables the inference of reticulate nodes (hybridization events). Due to computational limitations, for this analysis we reduced the taxon sampling to 12 representative species (two species from each clusioid family, and two outgroups), and used a set of 63 nuclear gene trees that could be rooted using Celastraceae as the outgroup. These nuclear trees were generated with RAxML‐NG v.1.2.0 (Kozlov et al. 2019) and rooted with *Stackhousia minima* Hook.f. (Celastrales) with the *pxrr* command in *Phyx* (Brown et al. 2017). Phylogenetic networks, allowing for one to four reticulations, were inferred under maximum pseudo‐likelihood, and five optimal phylogenetic networks were returned for each run. The command *CalGTProb* was used to compute the likelihood scores of the best network, scores that were then compared using a LRT. All phylogenetic networks were visualized using Dendroscope3 (Huson and Scornavacca 2012).

## 3 Results

### 3.1 The Clusioids626 Probe Set

Our nuclear data matrix includes 5290 target sequences, for a total size of 6,568,704 nucleotides, resulting in 39,936 probes padding 626 target genes. The median alignment length for these genes is 1742 bp (±1023.82 SD) and a median *P*_PIC_ of 0.14 (Table S5). The biotinylated probes have a mean alignment length of 111.43 bp, mean identity to the target data matrix of 96%, and mean coverage of the probes per target of 93.50%. The Clusioids626 kit targets three distinct sets of genes: (i) 273 nuclear genes singled out by MarkerMiner and not present among the Angiosperms353 targets; (ii) 150 Angiosperms353 targets not found by MarkerMiner; and (iii) 203 custom‐selected genes singled out by MarkerMiner and present in the Angiosperms353 panel.

### 3.2 Target Enrichment and Data Mining Performance

We produced novel TCS data for 36 species belonging to the clusioid clade plus one outgroup (*Linum suffruticosum* L.) that, together with the SRA data downloaded from NCBI, amounted to a total of 70 accessions. On average, 24.30 million paired reads were generated per sample and kept after quality filtering (ranging from 0.64 to 539.71 million reads; see heatmaps in Figure S1).

The percentage of on‐target reads varied across specimens, ranging from 0.20% to 86.10% (mean = 31%, median = 29.40%; Table 1, Figure S2), and families. Hypericaceae has the highest mean percentage of on‐target reads (56.34%), followed by Calophyllaceae (39.55%). Podostemaceae exhibits the lowest on‐target read‐recovery rate (2%; Table 1, Figure S2). In total, 608 of 626 targeted loci (97.12%) were recovered across the 70 specimens analysed (Figure S1, Table S6). A median of 481.50 loci were recovered at a 50% target length (mean = 395.30, min = 30, max = 608). The capture success rate was measured as the percentage of the total captured length of all target loci per individual relative to the total mean length of all loci. Please note that total captured length may exceed target length, meaning percentages can surpass 100%. The mean capture success rate was 110.40% (Table S6), ranging from 13.22% ( *Garcinia portoricensis* , Clusiaceae) to 201.70% (*Hypericum tomentosum*, Hypericaceae).

**TABLE 1:** Summary statistics of target capture sequencing (TCS) success, including total number of filtered raw reads (NumReads), percent of on‐target reads (PctOnTarget), the number of genes mapping to target (GenesMapped), and the number of loci retained at 50% target length (GenesAt50pct).

Family	Taxon	Name	NumReads	PctOnTarget	GenesMapped	GenesAt50pct
Hypericaceae	*Harungana madagascarensis*	C105	5,104,596	56.30	626	601
Hypericaceae	*Psorospermum senegalense*	C109	42,102,790	74.10	626	585
Calophyllaceae	*Lebrunia bushaie*	C1102	641,146	51.10	626	318
Bonnetiaceae	*Ploiarium elegans*	C1103	13,482,066	58.40	626	458
Hypericaceae	*Hypericum foliosum*	C114	11,182,924	82.30	626	607
Calophyllaceae	*Haploclathra paniculata*	C1141	6,794,246	52.20	626	526
Calophyllaceae	*Mesua ferrea*	C1142	32,875,932	82.90	626	557
Calophyllaceae	*Kayea nuda*	C1143	7,752,542	14.40	626	507
Calophyllaceae	*Clusiella elegans*	C1172	694,940	24.10	626	98
Calophyllaceae	*Poeciloneuron indicum*	C1174	18,264,412	35.50	626	598
Hypericaceae	*Hypericum galioides*	C133	20,169,360	66.60	626	530
Hypericaceae	*Eliea articulata*	C189	4,899,152	58.20	626	604
Hypericaceae	*Harungana rubescens*	C192	3,883,046	54.10	626	598
Hypericaceae	*Hypericum longistylum*	C301	8,564,486	86.10	626	607
Hypericaceae	*Hypericum calcicola*	C366	3,425,804	73.60	626	608
Hypericaceae	*Hypericum tomentosum*	C428	8,813,024	75.50	626	608
Hypericaceae	*Hypericum elodes*	C69	9,890,866	43.80	626	529
Hypericaceae	*Cratoxylum arborescens*	C702	4,865,406	53.20	626	589
Hypericaceae	*Cratoxylum formosum subsp. formosum*	C704	3,398,946	42.60	626	581
Hypericaceae	*Cratoxylum glaucum*	C705	4,791,328	61.20	626	591
Hypericaceae	*Cratoxylum formosum* subsp. *pruniflorum*	C706	3,756,756	43.70	626	586
Hypericaceae	*Vismia baccifera*	C708	2,114,140	42.20	626	540
Hypericaceae	*Vismia bilbergiana*	C709	4,022,110	45.90	626	575
Hypericaceae	*Vismia cayennensis*	C710	2,536,426	40.10	626	557
Hypericaceae	*Vismia guianensis*	C711	3,682,204	44.60	626	582
Calophyllaceae	*Calophyllum brasiliense*	C835	16,456,140	40.90	626	591
Clusiaceae	*Garcinia brasiliensis*	C837	1,268,102	14.50	626	194
Calophyllaceae	*Endodesmia calophylloides*	C839	9,633,928	74.80	626	552
Calophyllaceae	*Caraipa llanorum*	C841	7,003,404	73.60	626	507
Clusiaceae	*Symphonia globulifera*	C846	4,007,402	18	626	259
Bonnetiaceae	*Bonnetia sessilis*	C848	2,618,940	4.50	626	92
Clusiaceae	*Moronobea riparia*	C849	5,665,994	49.30	626	199
Calophyllaceae	*Kielmeyera coriacea*	C851	4,479,618	76.60	626	458
Calophyllaceae	*Mammea vatoensis*	C852	4,457,186	62.50	626	554
Calophyllaceae	*Marila laxiflora*	C853	6,811,474	35	626	597
Calophyllaceae	*Neotatea longifolia*	C854	16,508,018	32.40	626	597
Linaceae	*Linum suffruticosum*	C947	2,609,744	20	625	200
Podostemaceae	*Dalzellia ubonensis*	DRR238799	51,026,638	2.90	607	522
Podostemaceae	*Hydrobryum japonicum*	DRR238809	40,302,216	1.40	590	486
Podostemaceae	*Polypleurum stylosum*	DRR238819	36,182,322	3.90	588	477
Podostemaceae	*Terniopsis brevis*	DRR238825	101,763,126	2.20	618	537
Podostemaceae	*Weddellina squamulosa*	DRR238827	42,205,222	2.20	610	516
Podostemaceae	*Zeylanidium tailichenoides*	DRR258785	84,168,300	0.30	576	203
Linaceae	*Linum tenue*	ERR10035165	17,142,584	4.10	624	250
Clusiaceae	*Tovomita fructipendula*	ERR10978005	1,255,758	41	586	93
Calophyllaceae	*Mammea americana*	ERR2040359	16,211,672	2.90	625	324
Clusiaceae	*Garcinia oblongifolia*	ERR2040362	16,269,602	4.20	621	418
Clusiaceae	*Garcinia livingstonei*	ERR2040363	27,122,088	1.60	617	339
Ochnaceae	*Ochna serrulata*	ERR2040388	23,604,924	2.40	607	380
Ochnaceae	*Cespedesia spathulata*	ERR5034039	3,339,896	7.10	611	152
Ochnaceae	*Godoya obovata*	ERR5034041	2,562,200	7.50	603	152
Ochnaceae	*Brackenridgea zanguebarica*	ERR5034068	3,332,182	9.30	619	129
Ochnaceae	*Campylospermum reticulatum*	ERR5034069	1,699,686	3.40	594	115
Ochnaceae	*Fleurydora felicis*	ERR5034071	2,445,734	8.80	603	141
Celastraceae	*Elaeodendron orientale*	ERR7618728	5,193,870	8.50	562	130
Celastraceae	*Stackhousia minima*	ERR7619745	6,571,210	6.70	597	94
Hypericaceae	*Cratoxylum cochinchinense*	ERR7621010	6,267,036	26.40	624	180
Calophyllaceae	*Kielmeyera petiolaris*	ERR7621021	1,898,258	37.10	609	162
Calophyllaceae	*Caraipa densifolia*	ERR7621022	2,802,468	35.30	614	131
Clusiaceae	*Moronobea coccinea*	ERR7621023	5,410,120	36.90	605	175
Celastraceae	*Tripterygium wilfordii*	SRR12059219	62,865,170	2.60	625	420
Podostemaceae	*Marathrum utile*	SRR12956182	539,716,186	1.30	616	503
Clusiaceae	*Garcinia anomala*	SRR14356990	75,830,742	0.20	625	213
Calophyllaceae	*Calophyllum longifolium*	SRR15016251	19,854,228	3.60	626	504
Bonnetiaceae	*Bonnetia paniculata*	SRR16214477	46,248,790	0.20	626	46
Calophyllaceae	*Calophyllum inophyllum*	SRR17138441	41,340,556	0.40	626	30
Clusiaceae	*Garcinia portoricensis*	SRR19237490	49,098,458	4.30	626	555
Linaceae	*Linum usitatissimum*	SRR20318611	46,938,580	2.20	622	340
Calophyllaceae	*Calophyllum macrocarpum*	SRR7412626	3,849,732	16.20	625	242
Clusiaceae	*Clusia rosea*	SRR7412628	7,447,036	7.10	626	372
MEDIAN				29.40	626	481.50
MEAN				30.84	618.77	395.30

The median taxon occupancy per target locus was 56 species, with a median alignment length of 1860 bp (±1465.65 SD) and 0.41 *P*_PIC_ (Table S7), more than double the *P*_PIC_ value for the probe set target sequences alone (*P*_PIC_ = 0.14; Table S5). Nineteen genes were discarded because their coverage scores (max_overlap script) were < ⅔ of the median; six of these were Angiosperms353 targets. Additionally, six target loci were flagged for potential paralogs (≥ 3 variants); three were Angiosperms353 targets and were excluded from downstream analyses. After outlier removal and matrix refinement, 552 nuclear MSAs were generated, with a median alignment length of 1271.50 bp (447–6500 bp) and a *P*_PIC_ of 0.49 (0.27–0.66). The concatenated nuclear data matrix had a size of 767,230 bp.

### 3.3 Probe Sets Comparison

When processing our dataset with the Angiosperms353 target file, HybPiper recovered 343 genes (97.16% of targets) at a 50% target length across the 70 samples analysed, with a median of 198 loci (mean = 190, min = 1, max = 343; Figure S1, Table S8). The percentage of on‐target reads ranged from 0% to 18.50%, with a median value of 5.35% (mean = 4.85%) and with median taxon occupancy per locus of 56 taxa. MSAs lengths ranged from 192 to 4892 bp, with a mean length of 956.96 bp (Tables 2, S9). In contrast, when processing our dataset with the Clusioids626 target file, the MSA lengths ranged from 351 to 12,092 bp, with an average MSA length of 2135.78 bp (Tables 2, S7).

**TABLE 2:** Number of taxa, length of the aligned contigs, percent of missing data, and number of Parsimony‐informative characters (PIC) for two different datasets: (A) Aligned contigs of the loci targeted with the Clusioids626 probe set, and (B) aligned contigs of the loci targeted with the Angiosperms353 enrichment panel.

	No of taxa	Alignment length	Missing percent	Parsimony‐informative characters
*(A) Clusioid626 (608 genes)*				
Mean	55.67	2135.78	45.54	776.82
SD	6.49	1465.65	15.09	401.73
Max	69	12,092	83.60	3097
Min	36	351	4.41	160
Total	70	1,298,556		472,309
*(B) Angiosperms353 (343 genes)*				
Mean	44.87	956.96	27.63	423.90
SD	18.70	651.19	14.08	254.89
Max	67	4892	75.73	1450
Min	13	192	1.69	89
Total	70	330,151		146,246

Regardless of the target file used to process the data, the larger the MSA length the greater the number of Parsimony‐informative characters (PICs); however, the highest PIC values were obtained for data processed with the Clusioids626 target file (Figure S3). Regarding taxon occupancy (Table 2), it was higher for the Clusioids626 target file (mean = 55.67 taxa, min = 36, max = 69) than for the Angiosperms353 target file (mean = 44.87 taxa, min = 13, max = 67). The percent of capture success was highest for genes mined with the Clusioids626 target file (110.40%), recovering an average of 483.56 loci (77.25% of all Clusioids626 targets; Table S6); whereas, for data generated with the Angiosperms353 target file, capture success was lower (51.68%) and we recovered an average of 226.61 loci (64.12% of all Angiosperms353 targets; Table S6).

We evaluated topological support, resolution, and discordance for the MSC nuclear trees generated with sequences assembled either with the Clusioids626 or the Angiosperms353 target files. For each analysis, we calculated normalized quartet scores (QS), the effective number of genes per branch/node (EN), the total number of quartets in all the gene trees supporting the main topology (q1), and local posterior probabilities (LPPs; Table S10). In the Clusioids626 MSC nuclear phylogeny, the proportion of gene trees per node supporting the primary topologies (q1) exceeded ⅓ for 67 nodes, 0.50 for 52 nodes, and 0.75 for 35 nodes (Figure S4). Similarly, Angiosperms353 q1 showed a gene tree proportion > ⅓ for 64 nodes, 0.50 for 49 nodes, and 0.75 for 35 nodes (Figure S4). In both cases, the mean proportion of gene trees per node supporting q1 was 0.73. The Clusioids626 MSC phylogeny showed 45 nodes, out of 66 nodes, with maximum support (LPP = 1), 17 with high support (1.0 > LPP ≥ 0.9), three nodes with moderate support (0.9 > LPP ≥ 0.7), and two of them with low support (LPP < 0.7). On the other hand, the Angiosperms353 MSC tree showed 40 out of 64 nodes with maximum support, 17 with high support, five with moderate support, and five with low support (Table S10).

### 3.4 Plastid Data Matrix Assembly

We successfully recovered sequences from 105 plastid loci from off‐target reads (Table S11), achieving a mean taxon occupancy of 39 taxa, a mean alignment length of 1328 bp (90–22,892 bp), and a *P*_PIC_ of 0.24 (Table S12). Following the calculation of coverage scores with the max_overlap script, 14 taxa were excluded from the plastid dataset (median coverage score < ⅓). Similarly, 11 plastid CDS were discarded (median coverage score < ⅔). Additionally, five plastid regions were flagged by HybPiper as having potential paralogs (≥ 3 variants) and were excluded from downstream analysis. Once outlier removal and matrix optimization were completed, MSAs were inferred for 64 plastid CDSs. These MSAs had a median alignment length of 591 bp (170–5759 bp) and a *P*_PIC_ of 0.24 (0.10–0.71). The total alignment length of the concatenated plastid data matrix is 60,498 bp.

### 3.5 Phylogenomic Inference

**FIGURE 1:**
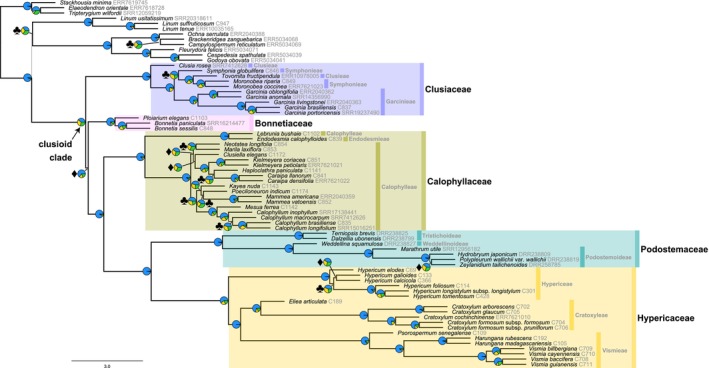
Nuclear species tree inferred under the multispecies coalescent (MSC) in ASTRAL‐III, for 70 clusioid‐clade and related taxa, from 552 targets assembled with our Clusioids626 target file. The thickness of the branches is proportional to the support (local posterior probabilities, LPPs). Pies charts on branches represent normalized quartet score (QS) values for each alternative topology (blue = species tree topology (q1); green = first alternative topology; yellow = second alternative topology). Diamonds (♦) beside pie charts indicate nodes where incomplete lineage sorting (ILS) could be detected, whereas club symbols (♣) indicate evidence of introgression.

Nuclear species trees were strongly supported for both MSC and MLE analyses (Figure 1, Figures S5 and S6). Both the MSC and MLE nuclear topologies support the monophyly of the clusioid clade and its five constituent families. Trees were rooted with Celastrales. The MSC nuclear species tree was inferred from 552 MLE gene trees, with bipartitions collapsed for SH‐aLRT support values = 0 (collapsing under various bootstrap thresholds did not alter the species tree topology but affected support values). Navigating the topology from the tips towards the root, the nuclear phylogeny recovered a Podostemaceae plus Hypericaceae clade sister to Calophyllaceae, with these three families sister to Bonnetiaceae, and all of them sister to Clusiaceae (LPP = 1). The MLE nuclear tree derived from the analysis of the concatenated dataset had maximal UFB and SH‐aLRT support values (100%) across most branches (Figure S6). This concatenated MLE tree recovered the same topology as the MSC nuclear species tree. A nuclear topology from data mined using the Angiosperms353 target file alone was inferred under the MSC framework. This MSC Angiosperms353 tree recovered the same overall topology as the MLE and MSC nuclear trees obtained from the Clusioids626 targets. However, some unsupported variation was observed within family Calophyllaceae, especially pertaining to genera *Clusiella*, *Marila*, and *Neotatea* (Figure S7).

**FIGURE 2:**
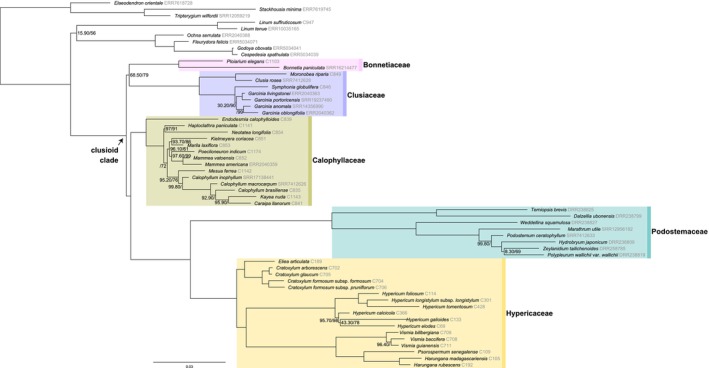
Maximum likelihood estimation (MLE) plastid phylogeny inferred in IQ‐TREE2, for 57 clusioid‐clade and related taxa, from 64 concatenated and partitioned plastid coding DNA sequences (CDS) mined with our custom amino acid plastid target file. Branch support shown for ultrafast bootstrap (UFB) and SH‐like approximate likelihood ratio tests (SH‐aLRT) if support value < 100.

**FIGURE 3:**
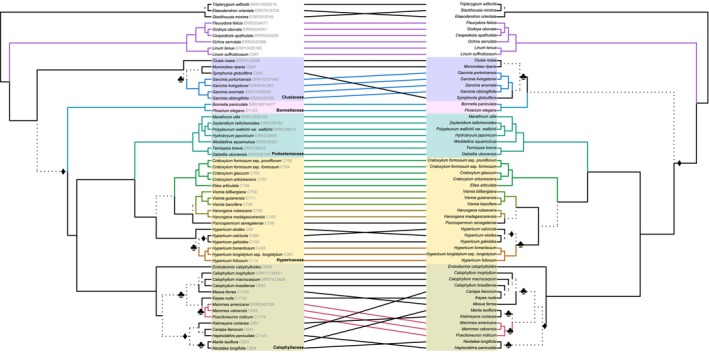
Tanglegram of the clusioid clade and related taxa comparing ultrametric topologies derived from the MSC nuclear (left) and MLE plastid (right) trees. Dashed lines highlight a combination of tips just present in one topology (i.e., unique clades). Subtrees shared across topologies are similarly coloured. Diamonds (♦) indicate nodes where incomplete lineage sorting (ILS) could be detected, whereas club symbols (♣) indicate nodes with evidence of introgression.

The concatenated plastid data matrix, spanning 57 samples and 67 CDS, also yielded high support values (UFB/SH‐aLRT > 90%). The MLE topology inferred from these data was largely congruent with the nuclear phylogeny but exhibited some discordance, primarily along internal nodes (Figures 2 and 3). Family Bonnetiaceae was sister to Clusiaceae with moderate support (UFB = 68.60%/SH‐aLRT = 79%), and both were sister to a clade composed of Calophyllaceae sister to Podostemaceae plus Hypericaceae (UFB/SH‐aLRT = 100%). Within Clusiaceae, conflict was detected regarding the placement of *Moronobea* Aubl., which was sister to *Clusia rosea* Jacq. in the plastid tree (UFB/SH‐aLRT = 100%, Figure 3). Calophyllaceae exhibits the highest conflict between nuclear and plastid phylogenies (Figure 3), with six out of 11 analysed genera (*Caraipa*, *Haploclathra*, *Kayea*, *Kielmeyera*, *Marila*, and *Neotatea*) changing their placement across topologies. These shifts were supported by high bootstrap values in most cases (> 90%; Figures 2 and 3). Within Hypericaceae, conflict pertains to the placement of the tribes Cratoxyleae, Hypericeae, and Vismieae. The MLE plastid tree recovered tribe Cratoxyleae as sister to a Hypericeae and Vismieae clade (Figures 2 and 3), while the MSC and MLE nuclear species trees recovered Hypericeae as sister to a Cratoxyleae and Vismieae clade, in all cases with maximum support. The nuclear and plastid topologies were identical for tribe Cratoxyleae, which was not the case for tribes Hypericeae and Vismieae (Figures 2 and 3). In the plastid MLE tree, within tribe Hypericeae, *Hypericum elodes* L. was sister to *H. galioides* Lam. (UFB = 43.80%, SH‐aLRT = 78%, Figure 2). Meanwhile, within tribe Vismieae, conflict was detected regarding the placement of *Vismia* (UFB/SH‐aLRT = 100%, Figures 2 and 3).

To explore the evolutionary processes that may underlie this discordance, we examined alternative quartet frequencies (QS values), focusing on the second (q2) and third (q3) alternative quartet topologies obtained from ASTRAL‐III for the nuclear MSC tree (Table S10). Across the phylogeny, we found a strong signal (QS ≥ ⅓ for at least two alternative quartets) of gene tree discordance for 14 nodes (Figure 1). Similar proportions of trees supporting q2 and q3 suggest ILS, within *Hypericum* ( *H. galioides* –*H. calcicola* divergence), Podostemoideae (*Polypleurum wallichii* var. *wallichii*–*Zeylanidium tailichenoides*), two deep Calophyllaceae nodes (spanning six genera, i.e., *Neotatea*, *Marila*, *Clusiella*, *Kielmeyera*, *Haploclathra*, *Caraipa*), and the most recent common ancestor (MRCA) of the clusioid clade, excluding Clusiaceae. The results also suggest multiple introgression events (asymmetric q2 and q3 frequencies), primarily within Hypericeae (five, out of six, *Hypericum* spp.) and Clusiaceae (*Symphonia*–*Tovomita*–*Moronobea* clade). Notably, the majority of inferred introgression events occur within family Calophyllaceae (Table S10). When focussing on the nodes showing nuclear vs. plastid incongruence (Figure 3), seven nodes exhibit a strong signal of nuclear gene tree discordance (see threshold above). Three of these nodes exhibit similar q2 and q3 frequencies, consistent with ILS, i.e., the *H. galioides* and *H. calcicola* clade, the node including all clusioid families, except for Clusiaceae, and the deep Calophyllaceae node above. Conversely, four nodes display asymmetric q2 and q3 frequencies, suggesting introgression, i.e., the Hypericeae clade (excluding *H. elodes*), the Symphonieae‐Clusieae clade (Clusiaceae), and two Calophyllaceae nodes (the *Neotatea*‐*Marila* and *Kayea*‐*Mesua* clades).

### 3.6 Reticulation: Splits and Phylogenetic Networks

**FIGURE 4:**
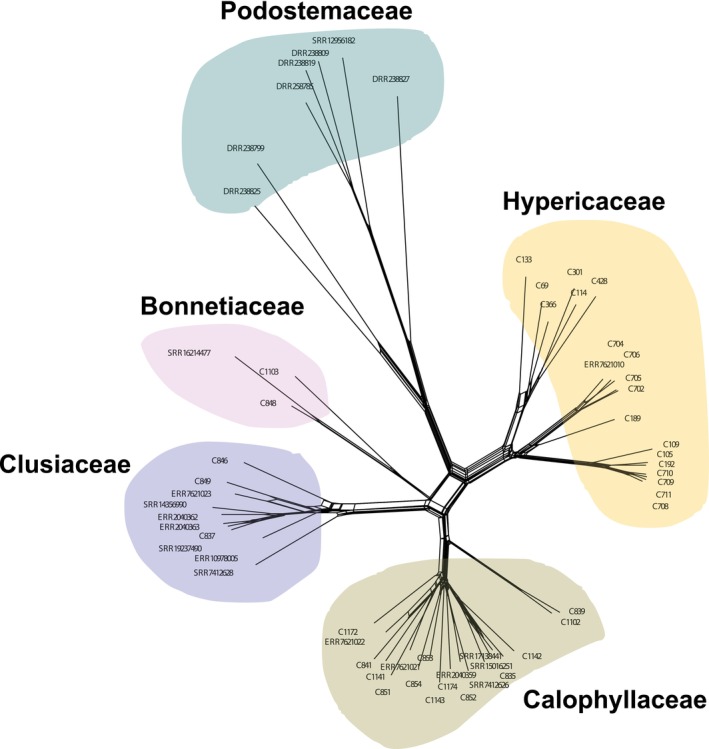
Nuclear splits network of the clusioid clade, inferred in SplitsTree (using the Neighbour‐Net method, with uncorrected P‐distances) from 552 concatenated, partitioned genes. Colouring scheme for families as in Figures 1 and 2.

The SplitsTree network (Figure 4) resolved the five clusioid families into five distinct, well‐supported groups (most bootstrap support values > 80%), consistent with the phylogenetic trees. This splits network showed parallel edges at deep branches across all families, suggesting ancestral reticulation. Calophyllaceae and Podostemaceae exhibited the highest density of parallel edges at their stem. Clusiaceae displayed the longest parallel edges at its crown, reflecting extensive reticulation, and Bonnetiaceae showed the fewest parallel edges. Tip branches were longest in Podostemaceae.

We further investigated the signals of introgression using PhyloNet. The PhyloNet analysis recovered a topology similar to the MSC species tree (Figure S8). The best‐fit phylogenetic network (log likelihood = −379.16) had four reticulations. This network indicates possible gene flow between the ancestor of *Linum suffruticosum* and an unsampled/extinct lineage ancestral to Podostemaceae. Within Podostemaceae, *Marathrum utile* Tul. and *Terniopsis brevis* M.Kato could have resulted from hybridization between unsampled/extinct lineages ancestral to both Podostemaceae and Hypericaceae. *Ploiarium elegans* Korth. (Bonnetiaceae) could have resulted from hybridization between unsampled/ancestral lineages to *Bonnetia sessilis* Benth. and family Calophyllaceae.

## 4 Discussion

Hyb‐Seq has revolutionized the study of plant systematics (Dodsworth et al. 2019) by providing high‐resolution data at multiple evolutionary scales and from DNA material of varying quality (Zuntini et al. 2024; Balant et al. 2025). The latest Hyb‐Seq advancement pertains the integration of phylogenetically‐broad enrichment panels (e.g., Angiosperms353, Johnson et al. 2019) with lineage‐specific probe sets (e.g., Fonseca et al. 2023, 2024), enabling the development of custom kits that offer high resolution within groups, while retaining broad applicability across lineages.

### 4.1 The Clusioids626 Probe Set

Here we introduce the development and implementation of the Clusioids626 probe set, a novel tool that combines universal and custom probes to recover up to 626 nuclear ortholog genes chosen to resolve phylogenetic relationships across the clusioid clade. This kit is tailored to capture genetic data from all clusioid families (Bonnetiaceae, Calophyllaceae, Clusiaceae, Hypericaceae, and Podostemaceae), while also incorporating the universal Angiosperms353 targets. Unlike most existing combined kits, which are typically designed for the family‐level or below (Bentz and Leebens‐Mack 2024; Eserman et al. 2021; Fonseca et al. 2023, 2024; Musker et al. 2025; Ogutcen et al. 2021), the Clusioids626 kit is the only one developed for the supra‐familial level. This strategy removes the need for separate kits for each clusioid family while maintaining the high resolution of lineage‐specific probe sets and the broad applicability of universal enrichment panels. Clusioids626 is also among the few probe sets specifically designed for malpighialean lineages. To date, three other custom kits have been designed within this megadiverse order, targeting families Euphorbiaceae (431 loci, Villaverde et al. 2018), Ochnaceae (275 loci, Schneider et al. 2021; Shah et al. 2022), and Salicaceae (972 loci, Sanderson et al. 2020), and none of them incorporate the general‐purpose Angiosperms353 targets.

Probe sets combining Angiosperms353 with custom targets have, on average, lower percentages of on‐target reads (e.g., 30% for the Asparagaceae1726 kit, Bentz and Leebens‐Mack 2024) than custom probe sets without universal targets (e.g., 86.70% for the Salicaceae972 kit, Sanderson et al. 2020). This pattern seems to reflect a trade‐off between achieving broad taxonomic applicability at the cost of slightly reduced hybridization efficiency for certain taxa, which has been reported at supra‐familial levels (Liu et al. 2019). However, samples processed with our Clusioids626 probe set yielded on average 50.40% on‐target reads. This value is comparable to the average capture efficiency detected for combined kits (e.g., 49.50% for the Annonaceae799 kit, Fonseca et al. 2024), and for some specific kits within Malpighiales, such as the Euphorbiaceae431 kit (48.60%, Villaverde et al. 2018). Nonetheless, it is not as high as the average for the Salicaceae972 specific kit (see above). Differences in target recovery across studies may also be due to variations in molecular protocols (e.g., DNA purification, genomic library preparation, pooling strategies, hybridization settings) and computational workflows (e.g., mapping approach, target file sequence composition; Andermann et al. 2020).

Given our on‐target recovery percent (50.40%), our probe set successfully captures a broad range of nuclear orthologs, providing sufficient phylogenetic signal for robust macroevolutionary analyses across the clusioid clade (see below). Its applicability at intraspecific levels remains to be explored, but we expect the Clusioids626 probe set to outmatch the Angiosperms353 enrichment panel at the microevolutionary scale, as it contains most Angiosperms353 targets plus 273 clusioid‐specific ones. Beyond clusioid lineages, we were also able to mine most of our Clusioids626 targets (using our nuclear target file) for other malpighialean lineages (e.g., Linaceae, Ochnaceae) and even for taxa outside the order (i.e., three Celastrales species; Table 1). As expected, capture success decreases with increasing phylogenetic distance from the targets, indicating that the transferability of the probe set is highest within the clusioid clade and progressively reduced for more distant malpighialean lineages. Nonetheless, we were still able to mine 94–420 genes for the order Celastrales (from NCBI SRAs) using our nuclear Clusioids626 target file. This suggests modifying the hybridization settings (e.g., lowering the temperature down to 62.5°C) should result in increased capture success for other closely‐related angiosperm groups (e.g., across the Malvids), provided sequencing depth is sufficient (see myBaits Manual v.5.02). Overall, these findings indicate that the Clusioids626 probe set is an efficient and adaptable tool for phylogenomics and, putatively, it should be of use for population genomics research, within the clusioid clade. Clusioids626 thus offers a distinct advantage: by operating at an intermediate taxonomic level and combining universal and specific probes, it balances efficiency, specificity, and broad applicability. Furthermore, off‐target reads provide valuable genetic information (i.e., organellar DNA) to further support evolutionary inference for clusioid Malpighiales (see below).

### 4.2 Clusioids626 Versus Angiosperms353

We show here that a combined kit, mixing universal and specific probes, offers additional advantages over universal kits by themselves. The custom Clusioids626 probe set outperforms the universal Angiosperms353 enrichment panel in gene recovery and capture success for the clusioid clade (Tables S6 and S8). This is consistent with other studies showing that lineage‐specific probe sets improve locus recovery by targeting orthologs with higher affinity in a given clade (Ufimov et al. 2021). The inclusion of universal Angiosperms353 loci ensures compatibility with existing datasets, facilitating broader comparative analyses (Johnson et al. 2019; Baker et al. 2022; Zuntini et al. 2024), while the custom loci improve resolution for lineage‐specific relationships by increasing variable sites (1094.16 in Clusioids626 vs. 541.26 in Angiosperms353) and PICs (776.82 Clusioids626 vs. 423.90 Angiosperms353) median values. Despite comparable median values of *P*_PIC_ per aligned sequence length (0.36 for Clusioids626 vs. 0.44 for Angiosperms353), the Clusioids626 probe set resulted in more robust topological support values, with more nodes achieving maximum LPP (Table S10) and with a higher proportion of nodes resolving gene‐tree conflict (QS > ⅓). Notably, both kits recovered a similar number of nodes achieving QS values > 0.75, indicating the power of both targeted sequencing kits to resolve phylogenetic relationships within the clusioid clade.

Previous studies (Chau et al. 2018; Larridon et al. 2020; Siniscalchi et al. 2021; Fonseca et al. 2024) also suggest that universal loci (e.g., Angiosperms353) provide comparable phylogenetic resolution to taxon‐specific targets, but highlight a critical advantage of custom kits: notably improved capture success and variable site recovery. This is also true for our Clusioids626 kit. Our results evidence that custom probe sets improve the quantity and quality of data, enabling more robust phylogenomic (and putatively population genomic) analyses by increasing the number of informative loci (and maybe even SNPs). Thus, while universal kits remain valuable for broad comparisons, custom optimizations offer a strategic advantage for clade‐specific studies.

### 4.3 Clusioid Clade Phylogenomics

The Clusioids626 probe set was applied to resolve phylogenetic relationships among and within families in the clusioid clade. For the first time here, we place in a phylogeny eight clusioid species for which there was no sequence data available. Furthermore, we generate additional data for 36 clusioids and one Linaceae species. Relationships inferred in this study, for 58 species representing all five clusioid families, all eight clusioid tribes, and 34 of the 92 genera currently recognized in the group, are mostly resolved and strongly supported, both for the nuclear (Figure 1, Figures S5 and S6) and plastid (Figures 2 and 3) trees.

We confirm, in both the nuclear and plastid topologies, the monophyly of the clusioid clade with maximum support (Cai et al. 2019, 2021; Ruhfel et al. 2011; Zuntini et al. 2024). Within the clusioid clade, our nuclear and plastid trees both recover Calophyllaceae as sister to Hypericaceae plus Podostemaceae, in line with previous findings (Cai et al. 2019; Ramírez‐Barahona et al. 2020; Ruhfel et al. 2011; Sun et al. 2016; Xi et al. 2012). The placement of Podostemaceae within the clusioid clade has been recently disputed by Baker et al. (2022) in their order‐level analysis of angiosperms, placing Podostemaceae outside Malpighiales, perhaps as a result of a long‐branch attraction (LBA) artefact in their massive data matrix (but see Zuntini et al. 2024). It is quite remarkable that subtending long branches characterize the backbone of Podostemaceae in our study (Figures 1 and 2, Figures S5 and S6) and previous ones (Ramírez‐Barahona et al. 2020; Li et al. 2021; although see Tippery et al. 2011; Philbrick et al. 2018; Bedoya et al. 2019). Meanwhile, branches within Podostemaceae subfamilies (Podostemoideae, Tristichoideae, and Weddellinoideae) are much shorter, suggesting fewer changes have accumulated since the MRCA of the sampled species diverged (Soltis et al. 2019). The longer branches leading to Podostemaceae compared to other clusioids could indicate faster rates of evolution in their plastomes, as suggested by Ruhfel et al. (2016) and Bedoya et al. (2019), which could be attributed to a faster life cycle and shorter generations (Smith and Donoghue 2008); Podostemaceae is mainly represented by annual herbs that rely on water levels to complete their life cycle. Other aquatic lineages, such as Hydrostachyaceae (order Cornales), exhibit long branches as well, which could also result in LBA artefacts (Fan and Xiang 2003; but see Thomas et al. 2021). Faster mutation rates have also been associated with life‐history traits, such as plant height, genome size, and age at first reproduction (Lehtonen and Lanfear 2014; Bromham et al. 2015), and with pseudogenization following gene loss, that leads to a release of selective constraints (and hence faster mutation rates; Peredo et al. 2013; Li et al. 2023). The factors driving fast molecular evolution in Podostemaceae remain to be explored.

### 4.4 Cyto‐Nuclear Discordance in the Clusioid Clade

Topological incongruence between genomic compartments in our study and previous ones, as expected, centres around placements that have posed problems for a long time and that remain unsolved (Figures 1, 2, 3 and Figure S6). Specifically, the placement of families Bonnetiaceae and Clusiaceae, with respect to the remaining clusioid families. The backbone of our nuclear topology presents Clusiaceae as sister to all other clusioid families (Figure 1), in agreement with the results of Ramírez‐Barahona et al. (2020). However, our plastid tree recovers Clusiaceae sister to Bonnetiaceae (Figures 2 and 3), in agreement with other studies based on plastome‐level data (Xi et al. 2012; Trad et al. 2021) or on a few plastid and nuclear markers (Wurdack and Davis 2009; Ruhfel et al. 2011, 2016; Sun et al. 2016).

Some of the relationships identified for the nuclear compartment in our study contrast with those reported in previous studies relying on TCS. Specifically, Cai et al. (2021) placed Hypericaceae as sister to all other clusioid families and Clusiaceae as sister to a Bonnetiaceae and Calophyllaceae clade, based on 423 ultra‐conserved‐like elements (ULEs; Buddenhagen et al. 2016). It should be noted that the ultra‐conserved‐like nature of these targets limits the phylogenetic signal they might present for phylogenetic inference (vital for complex plant lineages, such as the malpighialean angiosperms). Nuclear species trees inferred from Angiosperms353 targets (Baker et al. 2022; Zuntini et al. 2024) have previously placed Bonnetiaceae as sister to the remaining clusioid families with full support.

Apart from methodological aspects potentially explaining topological incongruences between our study and previous ones, the degree of conflict revealed for clusioid clade families might stem from biological processes such as ILS, duplication (with or without loss), and/or hybridization (Larson et al. 2026) (see below), suggesting that the phylogenetic reconstruction of the clusioid clade is a major challenge, in line with the difficulties encountered in the reconstruction of Malpighiales as a whole (Cai et al. 2021).

### 4.5 Reticulation in the Clusioid Clade

The overall concordance between nuclear MLE and MSC phylogenies underscores the robustness of our nuclear targets for resolving clusioid clade relationships. However, considerable cyto‐nuclear discordance (between nuclear and plastid trees) highlights divergent evolutionary histories across genomic compartments, a phenomenon increasingly recognized in angiosperms (Stull et al. 2020, 2023; Kandziora et al. 2022; Joyce et al. 2025).

The splits network (Figure 4) revealed widespread parallel edges at deep nodes across all families, suggestive of ancestral phylogenetic conflict during their divergence, particularly pronounced in Podostemaceae and Calophyllaceae. Deep reticulation could result from several causes, such as ILS during rapid diversification leading to conflicting phylogenetic signal (Kandziora et al. 2022), ancient hybridization and reticulation due to ecological introgression (Nge et al. 2021), allopolyploidization (Soltis et al. 2019), ecological and biogeographic factors, and even methodological artefacts (Steenwyk et al. 2023; Bjornson et al. 2024; Joyce et al. 2025). In the clusioid clade, Podostemaceae exhibits the longest terminal branches and the densest reticulation at the stem, consistent with an accelerated molecular evolution scenario associated with adaptation to extreme aquatic habitats (Jin et al. 2020). Clusiaceae shows the longest parallel edges at its crown, suggesting recurrent gene flow, possibly driven by ecological versatility (e.g., facultative CAM photosynthesis, epiphytic growth; Qiu et al. 2023; Kramml et al. 2026) and high species diversity (Cardoso et al. 2017), facilitating hybridization in neotropical niches. Bonnetiaceae exhibits minimal reticulation, probably reflecting a stable evolutionary trajectory (Ruhfel et al. 2011).

We detected both ILS and introgression at work in shaping the evolutionary history of the clusioid clade and its constituent lineages. The similar q2 and q3 frequencies detected across multiple deep and intermediate nodes, where QS ≥ ⅓ for at least two alternative quartets, are consistent with ILS as a major driver of phylogenetic incongruence, potentially during early lineage diversification. This pattern is especially evident in groups such as *Hypericum* and Podostemoideae, as well as deep nodes within Calophyllaceae, among others (Figure 1), suggesting that ancestral polymorphisms would have been retained through successive speciation events.

Meanwhile, the asymmetrical support for alternative q2 and q3 topologies towards terminal nodes points to a complementary role for introgression, particularly within Calophyllaceae, Hypericeae, and Clusiaceae, where gene flow may have contributed to shaping more recent diversification (Figure 1). The contrasting signals observed with regards to the nuclear‐plastid incongruence further reinforce this interpretation: while the similar q2 and q3 frequencies for *Hypericum* are compatible with ILS, the imbalance detected in Calophyllaceae is more indicative of introgression. The phylogenetic network (Figure S8), inferred with PhyloNet, identifies potential gene flow between ancestral Linaceae and Podostemaceae, as well as between Hypericaceae and Podostemaceae. These results align with the deep reticulations and parallel edges of the splits network, suggesting historical introgression during the clusioid radiation.

Together, these findings underscore the importance of considering both ILS and hybridization when interpreting phylogenomic discordance, as well as the need for integrative approaches combining universal and taxon‐specific loci capable of disentangling the relative contribution of these factors across different temporal and phylogenetic scales.

## Author Contributions

A.S.M., L.P., and I.V.‐M. conceived the study. A.S.M., I.M.‐I., and N.M.N. obtained the samples. I.V.‐M. performed bioinformatic analyses to identify the clusioid targets. I.V.‐M., I.M.‐I., and L.P. did molecular work and analysed the data; I.V.‐M., I.M.‐I., L.P., and A.S.M. wrote the manuscript, with feedback from N.M.N. A.S.M. provided funding. All authors approved the final version of the manuscript.

## Funding

This work was supported by the Atracción de Talento CAM program, 2019‐T1/AMB‐12648, by project PID2020‐120145GA‐I00 funded by MICIU/AEI/10.13039/50110001033 and by ERDF “A way of making Europe”, by a Fundación Ramón Areces postdoctoral research fellowship, BEVP35A7117, and by Ramon y Cajal Grant RYC2021‐034942‐I funded by MCIN/AEI/10.13039/501100011033 and by European Union “NextGenerationEU”/PRTR.

## Conflicts of Interest

The authors declare no conflicts of interest.

## Supporting information


**Figure S1:** Heatmap of the recovered sequence length of (A) the 626 genes targeted with the Clusioids626 probe set, and (B) the 353 genes targeted with the Angiosperms353 enrichment panel.
**Figure S2:** Boxplots showing (A) the percentage of on‐target reads and (B) the total number of Clusioids626 target loci recovered at 50% target length for each clusioid family.
**Figure S3:** Scatter plot showing the relationship between the size and number of Parsimony‐informative characters (PICs) for each of the 608 genes from the Clusioids626 probe set and 345 genes from the Angiosperms353 enrichment panel.
**Figure S4:** Quartet score (QS) frequencies for focal internal nodes of the ASTRAL species tree, inferred from shrunk, trimmed gene trees. (A) QS frequencies for the 67 nodes of the species tree obtained using the Clusioids626 target file. (B) QS frequencies for the 63 nodes of the species tree obtained using the Angiosperms353 target file. The main topology (q1) is shown in red, and the two alternative topologies (q2, q3) are shown in blue. Dotted lines indicate the 1/3 threshold.
**Figure S5:** Coalescent‐based species trees generated by ASTRAL‐III for 70 samples of clusioid clade and related taxa enriched with Clusioids626, based on 552 loci. Node values represent local posterior probabilities (LPPs) for the main topology and are equal to one unless otherwise noted.
**Figure S6:** Maximum likelihood estimation‐based phylogenetic reconstruction of the clusioid clade inferred using 552 loci concatenated and mined with the Clusioids626 target file. Values at the nodes represent bootstrap support and Shimodaira‐Hasegawa‐like approximate likelihood ratio tests (SH‐aLRT) values, respectively.
**Figure S7:** Phylogenetic reconstructions using ASTRAL of the relationships in the clusioid clade and related taxa for 66 accessions from 337 loci mined with the Angiosperms353 target file. Branch values represent local posterior probabilities (LPPs) for the main topology (q1).
**Figure S8:** PhyloNet estimation results for the simplified phylogenetic tree of the clusioid clade inferred with the InferNetwork_MPL method, based on 63 rooted nuclear gene trees. The blue edges denote the reticulation events identified with numbers next to the edges, denoting the inheritance probabilities.

**Table S1:** De novo assembled transcriptomes used to design the Clusioids626 probe set.
**Table S2:** List of 1637 low copy nuclear genes with information of presence of multiple copies in other angiosperms and their function in *Arabidopsis thaliana* .
**Table S3:** Currently accepted species of the clusioid clade and their closest allies included in this study.
**Table S4:** Samples used in the design of the custom amino acid plastid target file.
**Table S5:** Summary statistics of the 626 nuclear targets included in the Clusioids626 probe set.
**Table S6:** Summary of loci length captured for the 626 nuclear loci selected to design the kit used in this study and the 353 nuclear loci from the Angiosperms353 enrichment panel.
**Table S7:** Summary statistics of target loci mined with Clusioids626 kit.
**Table S8:** Summary statistics of sequencing success for the loci mined using the Angiosperms353 kit: number of raw reads obtained (NumReads), percentage of on‐target reads (PctOnTarget), number of genes obtained (GenesMapped), and number of loci retained at 50% target length (GenesAt50pct).
**Table S9:** Summary statistics of target loci mined using the Angiosperms353 kit.
**Table S10:** Normalized quartet score (QS), effective number of genes for each node (EN), total number of quartets in all the gene trees supporting the main topology (q1), and the local posterior probabilities (the posterior of the three topologies adds up to 1) for the main topology obtained for both MSC phylogenetic trees constructed using the Clusioids626 and Angiosperms353 kits. Bold text indicates nodes with a high signal of incongruence, that is, QS values of the three alternative topologies greater than 0.1, with at least one of these values greater than 0.3.
**Table S11:** Summary statistics of plastid coding DNA sequences (CDS) recovery across clusioid samples obtained from the off‐target fraction by mining sequences with the amino acid plastid target file.
**Table S12:** Summary statistics of plastid loci mined using the custom AA plastid target file.

## Data Availability

The raw reads are available in the NCBI Sequence Read Archive (accession no. PRJNA1284379). The intermediate files (trimmed alignments, gene and species trees, and matrices used for phylogenetic networks), the scripts, and the workflow can be found on figshare (https://figshare.com/s/052464d25042e82170f2).

## References

[men70173-bib-0001] Ametrano, C. G. , J. Jensen , H. T. Lumbsch , and F. Grewe . 2025. “Unfate: A Comprehensive Probe Set and Bioinformatics Pipeline for Phylogeny Reconstruction and Multilocus Barcoding of Filamentous Ascomycetes (Ascomycota, Pezizomycotina).” Systematic Biology: 740–757. 10.1093/sysbio/syaf011. 39953951 PMC12699997

[men70173-bib-0002] Andermann, T. , M. F. Torres Jiménez , P. Matos‐Maraví , et al. 2020. “A Guide to Carrying Out a Phylogenomic Target Sequence Capture Project.” Frontiers in Genetics 10: 1407. 32153629 10.3389/fgene.2019.01407 PMC7047930

[men70173-bib-0003] Andrews, S. 2010. “Fastqc: A Quality Control Tool for High Throughput Sequence Data.” *Babraham Bioinformatics* [Computer Software]. http://www.bioinformatics.babraham.ac.uk/projects/fastqc/.

[men70173-bib-0004] Angiosperm Phylogeny Group . 2016. “An Update of the Angiosperm Phylogeny Group Classification for the Orders and Families of Flowering Plants: APG IV.” Botanical Journal of the Linnean Society 181, no. 1: 1–20. 10.1111/boj.12385.

[men70173-bib-0005] Anisimova, M. , M. Gil , J. F. Dufayard , C. Dessimoz , and O. Gascuel . 2011. “Survey of Branch Support Methods Demonstrates Accuracy, Power, and Robustness of Fast Likelihood‐Based Approximation Schemes.” Systematic Biology 60, no. 5: 685–699. 21540409 10.1093/sysbio/syr041 PMC3158332

[men70173-bib-0006] Baker, W. J. , P. Bailey , V. Barber , et al. 2022. “A Comprehensive Phylogenomic Platform for Exploring the Angiosperm Tree of Life.” Systematic Biology 71, no. 2: 301–319. 33983440 10.1093/sysbio/syab035 PMC8830076

[men70173-bib-0007] Balant, M. , D. Vitales , Z. Wang , et al. 2025. “Integrating Target Capture With Whole Genome Sequencing of Recent and Natural History Collections to Explain the Phylogeography of Wild‐Growing and Cultivated *Cannabis* .” Plants, People, Planet 7: 1–18.

[men70173-bib-0008] Bankevich, A. , S. Nurk , D. Antipov , et al. 2012. “SPAdes: A New Genome Assembly Algorithm and Its Applications to Single‐Cell Sequencing.” Journal of Computational Biology 19, no. 5: 455–477. 22506599 10.1089/cmb.2012.0021 PMC3342519

[men70173-bib-0009] Beck, J. B. , M. L. Markley , M. G. Zielke , et al. 2021. “Are Palmer's Elm‐Leaf Goldenrod and the Smooth Elm‐Leaf Goldenrod Real? The Angiosperms353 Kit Provides Within‐Species Signal in *Solidago ulmifolia* s. l.” Systematic Botany 46, no. 4: 1107–1113.

[men70173-bib-0010] Bedoya, A. M. , B. R. Ruhfel , C. T. Philbrick , et al. 2019. “Plastid Genomes of Five Species of Riverweeds (Podostemaceae): Structural Organization and Comparative Analysis in Malpighiales.” Frontiers in Plant Science 10: 1035. 31481967 10.3389/fpls.2019.01035 PMC6710714

[men70173-bib-0011] Bentz, P. C. , and J. Leebens‐Mack . 2024. “Developing Asparagaceae1726: An Asparagaceae‐Specific Probe Set Targeting 1726 Loci for Hyb‐Seq and Phylogenomics in the Family.” Applications in Plant Sciences 12: E11597. 39360194 10.1002/aps3.11597 PMC11443443

[men70173-bib-0012] Bjornson, S. , H. Verbruggen , N. S. Upham , and J. L. Steenwyk . 2024. “Reticulate Evolution: Detection and Utility in the Phylogenomics Era.” Molecular Phylogenetics and Evolution 201: 108197. 39270765 10.1016/j.ympev.2024.108197

[men70173-bib-0013] Bolger, A. M. , M. Lohse , and B. Usadel . 2014. “Trimmomatic: A Flexible Trimmer for Illumina Sequence Data.” Bioinformatics 30, no. 15: 2114–2120. 24695404 10.1093/bioinformatics/btu170 PMC4103590

[men70173-bib-0014] Borowiec, M. L. 2016. “AMAS: A Fast Tool for Alignment Manipulation and Computing of Summary Statistics.” PeerJ 4: E1660. 26835189 10.7717/peerj.1660 PMC4734057

[men70173-bib-0015] Breinholt, J. W. , S. B. Carey , G. P. Tiley , et al. 2021. “A Target Enrichment Probe Set for Resolving the Flagellate Land Plant Tree of Life.” Applications in Plant Sciences 9, no. 1: E11406. 33552748 10.1002/aps3.11406 PMC7845764

[men70173-bib-0016] Brewer, G. E. , J. J. Clarkson , O. Maurin , et al. 2019. “Factors Affecting Targeted Sequencing of 353 Nuclear Genes From Herbarium Specimens Spanning the Diversity of Angiosperms.” Frontiers in Plant Science 10: 1102. 31620145 10.3389/fpls.2019.01102 PMC6759688

[men70173-bib-0017] Bromham, L. , X. Hua , R. Lanfear , and P. F. Cowman . 2015. “Exploring the Relationships Between Mutation Rates, Life History, Genome Size, Environment, and Species Richness in Flowering Plants.” American Naturalist 185, no. 4: 507–524. 10.1086/680052 25811085

[men70173-bib-0018] Brown, J. W. , J. F. Walker , and S. A. Smith . 2017. “Phyx: Phylogenetic Tools for Unix.” Bioinformatics 33, no. 12: 1886–1888. 28174903 10.1093/bioinformatics/btx063 PMC5870855

[men70173-bib-0019] Buddenhagen, C. , A. R. Lemmon , E. Moriarty Lemmon , et al. 2016. “Anchored Phylogenomics of Angiosperms I: Assessing the Robustness of Phylogenetic Estimates.” *bioRxiv*: 086298.

[men70173-bib-0020] Cai, L. , Z. Xi , A. M. Amorim , et al. 2019. “Widespread Ancient Whole‐Genome Duplications in Malpighiales Coincide With Eocene Global Climatic Upheaval.” New Phytologist 221, no. 1: 565–576. 30030969 10.1111/nph.15357 PMC6265113

[men70173-bib-0021] Cai, L. , Z. Xi , E. M. Lemmon , et al. 2021. “The Perfect Storm: Gene Tree Estimation Error, Incomplete Lineage Sorting, and Ancient Gene Flow Explain the Most Recalcitrant Ancient Angiosperm Clade, Malpighiales.” Systematic Biology 70, no. 3: 491–507. 33169797 10.1093/sysbio/syaa083

[men70173-bib-0022] Camacho, C. , G. Coulouris , V. Avagyan , et al. 2009. “BLAST+: Architecture and Applications.” BMC Bioinformatics 10: 1–9. 20003500 10.1186/1471-2105-10-421 PMC2803857

[men70173-bib-0023] Capella‐Gutiérrez, S. , J. M. Silla‐Martínez , and T. Gabaldón . 2009. “trimAl: A Tool for Automated Alignment Trimming in Large‐Scale Phylogenetic Analyses.” Bioinformatics 25, no. 15: 1972–1973. 19505945 10.1093/bioinformatics/btp348 PMC2712344

[men70173-bib-0024] Cardoso, D. , T. Särkinen , S. Alexander , et al. 2017. “Amazon Plant Diversity Revealed by a Taxonomically Verified Species List.” Proceedings of the National Academy of Sciences of the United States of America 114, no. 40: 10695–10700. 28923966 10.1073/pnas.1706756114 PMC5635885

[men70173-bib-0025] Chamala, S. , N. García , G. T. Godden , et al. 2015. “Markerminer 1.0: A New Application for Phylogenetic Marker Development Using Angiosperm Transcriptomes.” Applications in Plant Sciences 3, no. 4: 1400115. 10.3732/apps.1400115 PMC4406834 25909041

[men70173-bib-0026] Chan, A. P. , J. Crabtree , Q. Zhao , et al. 2010. “Draft Genome Sequence of the Oilseed Species *Ricinus communis* .” Nature Biotechnology 28, no. 9: 951–956. 10.1038/nbt.1674 PMC2945230 20729833

[men70173-bib-0027] Chau, J. H. , W. A. Rahfeldt , and R. G. Olmstead . 2018. “Comparison of Taxon‐Specific Versus General Locus Sets for Targeted Sequence Capture in Plant Phylogenomics.” Applications in Plant Sciences 6, no. 3: e1032. 29732262 10.1002/aps3.1032 PMC5895190

[men70173-bib-0028] Chen, S. , Y. Zhou , Y. Chen , and J. Gu . 2018. “Fastp: An Ultra‐Fast All‐In‐One FASTQ Preprocessor.” Bioinformatics 34, no. 17: I884–I890. 30423086 10.1093/bioinformatics/bty560 PMC6129281

[men70173-bib-0029] Cook, C. D. K. 1996. Aquatic Plant Book. SPB Academic Publishing.

[men70173-bib-0030] Cronquist, A. 1981. An Integrated System of Classification of Flowering Plants. Columbia University Press.

[men70173-bib-0031] Davis, C. C. , C. O. Webb , K. J. Wurdack , C. A. Jaramillo , and M. J. Donoghue . 2005. “Explosive Radiation of Malpighiales Supports a Mid‐Cretaceous Origin of Modern Tropical Rain Forests.” American Naturalist 165, no. 3: 36–65. 10.1086/428296 15729659

[men70173-bib-0032] De Smet, R. , K. L. Adams , K. Vandepoele , M. C. Van Montagu , S. Maere , and Y. Van de Peer . 2013. “Convergent Gene Loss Following Gene and Genome Duplications Creates Single‐Copy Families in Flowering Plants.” Proceedings of the National Academy of Sciences of the United States of America 110, no. 8: 2898–2903. 23382190 10.1073/pnas.1300127110 PMC3581894

[men70173-bib-0033] Dodsworth, S. , L. Pokorny , M. G. Johnson , et al. 2019. “Hyb‐Seq for Flowering Plant Systematics.” Trends in Plant Science 24, no. 10: 887–891. 31477409 10.1016/j.tplants.2019.07.011

[men70173-bib-0034] Doyle, J. J. , and J. L. Doyle . 1987. “A Rapid DNA Isolation Procedure for Small Quantities of Fresh Leaf Tissue.” Phytochemical Bulletin 19, no. 1: 11–15.

[men70173-bib-0035] Ernst, E. 2003. Hypericum: The Genus Hypericum. Taylor & Francis.

[men70173-bib-0036] Eserman, L. A. , S. K. Thomas , E. E. Coffey , and J. H. Leebens‐Mack . 2021. “Target Sequence Capture in Orchids: Developing a Kit to Sequence Hundreds of Single‐Copy Loci.” Applications in Plant Sciences 9, no. 7: E11416. 34336404 10.1002/aps3.11416 PMC8312744

[men70173-bib-0037] Fan, C. , and Q. Y. Xiang . 2003. “Phylogenetic Analyses of Cornales Based on 26S Rrna and Combined 26S Rdna‐Mat*k*‐Rbc*l* Sequence Data.” American Journal of Botany 90, no. 9: 1357–1372. 21659236 10.3732/ajb.90.9.1357

[men70173-bib-0038] Fleming, J. F. , A. Valero‐Gracia , and T. H. Struck . 2023. “Identifying and Addressing Methodological Incongruence in Phylogenomics: A Review.” Evolutionary Applications 16, no. 6: 1087–1104. 37360032 10.1111/eva.13565 PMC10286231

[men70173-bib-0039] Fonseca, L. H. M. , P. Asselman , K. R. Goodrich , et al. 2024. “Truly the Best of Both Worlds: Merging Lineage‐Specific and Universal Probe Kits to Maximize Phylogenomic Inference.” Applications in Plant Sciences 12: E11615. 39628541 10.1002/aps3.11615 PMC11610415

[men70173-bib-0040] Fonseca, L. H. M. , M. M. Carlsen , P. V. Fine , and L. G. Lohmann . 2023. “A Nuclear Target Sequence Capture Probe Set for Phylogeny Reconstruction of the Charismatic Plant Family Bignoniaceae.” Frontiers in Genetics 13: 1085692. 36699458 10.3389/fgene.2022.1085692 PMC9869424

[men70173-bib-0041] FSF . 2014. “Free Software Foundation, Inc. GNU Datamash.” https://www.gnu.org/software/datamash/.

[men70173-bib-0042] Galili, T. 2015. “Dendextend: An R Package for Visualizing, Adjusting and Comparing Trees of Hierarchical Clustering.” Bioinformatics 31, no. 22: 3718–3720. 26209431 10.1093/bioinformatics/btv428 PMC4817050

[men70173-bib-0043] Gaudeul, M. , P. Sweeney , and J. Munzinger . 2024. “An Updated Infrageneric Classification of the Pantropical Species‐Rich Genus *Garcinia* L. (Clusiaceae) and Some Insights Into the Systematics of New Caledonian Species, Based on Molecular and Morphological Evidence.” Phytokeys 239: 73–105. 38523734 10.3897/phytokeys.239.112563 PMC10960151

[men70173-bib-0044] Goodstein, D. M. , S. Shu , R. Howson , et al. 2012. “Phytozome: A Comparative Platform for Green Plant Genomics.” Nucleic Acids Research 40, no. 1: 1178–1186. 10.1093/nar/gkr944 PMC3245001 22110026

[men70173-bib-0045] Grabherr, M. G. , B. J. Haas , M. Yassour , et al. 2011. “Trinity: Reconstructing a Full‐Length Transcriptome Without a Genome From RNA‐Seq Data.” Nature Biotechnology 29, no. 7: 644–652. 10.1038/nbt.1883 PMC3571712 21572440

[men70173-bib-0046] Gustafsson, M. H. , V. Bittrich , and P. F. Stevens . 2002. “Phylogeny of Clusiaceae Based on *rbc*L Sequences.” International Journal of Plant Sciences 163, no. 6: 1045–1054.

[men70173-bib-0047] Hale, H. , E. M. Gardner , J. Viruel , L. Pokorny , and M. G. Johnson . 2020. “Strategies for Reducing Per‐Sample Costs in Target Capture Sequencing for Phylogenomics and Population Genomics in Plants.” Applications in Plant Sciences 8, no. 4: E11337. 32351798 10.1002/aps3.11337 PMC7186906

[men70173-bib-0048] Hoang, D. T. , O. Chernomor , A. Von Haeseler , B. Q. Minh , and L. S. Vinh . 2018. “UFBoot2: Improving the Ultrafast Bootstrap Approximation.” Molecular Biology and Evolution 35, no. 2: 518–522. 29077904 10.1093/molbev/msx281 PMC5850222

[men70173-bib-0049] Huson, D. H. , and D. Bryant . 2024. “The SplitsTree App: Interactive Analysis and Visualization Using Phylogenetic Trees and Networks.” Nature Methods 21, no. 10: 1773–1774. 39223398 10.1038/s41592-024-02406-3

[men70173-bib-0050] Huson, D. H. , and C. Scornavacca . 2012. “Dendroscope 3: An Interactive Tool for Rooted Phylogenetic Trees and Networks.” Systematic Biology 61, no. 6: 1061–1067. 22780991 10.1093/sysbio/sys062

[men70173-bib-0051] Hutter, C. R. 2021. “AstralPlane.” https://github.com/chutter/AstralPlane.

[men70173-bib-0052] Jin, D. M. , J. J. Jin , and T. S. Yi . 2020. “Plastome Structural Conservation and Evolution in the Clusioid Clade of Malpighiales.” Scientific Reports 10, no. 1: 9091. 32499506 10.1038/s41598-020-66024-7 PMC7272398

[men70173-bib-0053] Johnson, M. G. , E. M. Gardner , Y. Liu , et al. 2016. “HybPiper: Extracting Coding Sequence and Introns for Phylogenetics From High‐Throughput Sequencing Reads Using Target Enrichment.” Applications in Plant Sciences 4, no. 7: 1600016. 10.3732/apps.1600016 PMC4948903 27437175

[men70173-bib-0054] Johnson, M. G. , L. Pokorny , S. Dodsworth , et al. 2019. “A Universal Probe Set for Targeted Sequencing of 353 Nuclear Genes From Any Flowering Plant Designed Using K‐Medoids Clustering.” Systematic Biology 68, no. 4: 594–606. 30535394 10.1093/sysbio/syy086 PMC6568016

[men70173-bib-0055] Joyce, E. M. , A. N. Schmidt‐Lebuhn , H. K. Orel , et al. 2025. “Navigating Phylogenetic Conflict and Evolutionary Inference in Plants With Target‐Capture Data.” Australian Systematic Botany 38: SB24011.

[men70173-bib-0056] Junier, T. , and E. M. Zdobnov . 2010. “The Newick Utilities: High‐Throughput Phylogenetic Tree Processing in the UNIX Shell.” Bioinformatics 26, no. 13: 1669–1670. 20472542 10.1093/bioinformatics/btq243 PMC2887050

[men70173-bib-0057] Kadlec, M. , D. U. Bellstedt , N. C. Le Maitre , and M. D. Pirie . 2017. “Targeted NGS for Species Level Phylogenomics:“Made to Measure” or “One Size Fits All”?” PeerJ 5: E3569. 28761782 10.7717/peerj.3569 PMC5530999

[men70173-bib-0058] Kalyaanamoorthy, S. , B. Q. Minh , T. K. Wong , A. Von Haeseler , and L. S. Jermiin . 2017. “ModelFinder: Fast Model Selection for Accurate Phylogenetic Estimates.” Nature Methods 14, no. 6: 587–589. 28481363 10.1038/nmeth.4285 PMC5453245

[men70173-bib-0059] Kandziora, M. , P. Sklenář , F. Kolář , and R. Schmickl . 2022. “How to Tackle Phylogenetic Discordance in Recent and Rapidly Radiating Groups? Developing a Workflow Using *Loricaria* (Asteraceae) as an Example.” Frontiers in Plant Science 12: 765719. 35069621 10.3389/fpls.2021.765719 PMC8777076

[men70173-bib-0060] Katoh, K. , and D. M. Standley . 2013. “MAFFT Multiple Sequence Alignment Software Version 7: Improvements in Performance and Usability.” Molecular Biology and Evolution 30, no. 4: 772–780. 23329690 10.1093/molbev/mst010 PMC3603318

[men70173-bib-0061] Korotkova, N. , J. V. Schneider , D. Quandt , A. Worberg , G. Zizka , and T. Borsch . 2009. “Phylogeny of the Eudicot Order Malpighiales: Analysis of a Recalcitrant Clade With Sequences of the *Pet*d Group II Intron.” Plant Systematics and Evolution 282: 201–228.

[men70173-bib-0062] Kozlov, A. M. , D. Darriba , T. Flouri , B. Morel , and A. Stamatakis . 2019. “RAxML‐NG: A Fast, Scalable and User‐Friendly Tool for Maximum Likelihood Phylogenetic Inference.” Bioinformatics 35, no. 21: 4453–4455. 31070718 10.1093/bioinformatics/btz305 PMC6821337

[men70173-bib-0063] Kramml, H. M. , J. B. Herpell , C. Priemer , et al. 2026. “ *Clusia* Genomes Shed Light on the Evolution and Diversity of Crassulacean Acid Metabolism Physiotypes.” Nature Communications 17: 3937. 10.1038/s41467-026-71958-z PMC13144421 42086566

[men70173-bib-0064] Larridon, I. , T. Villaverde , A. R. Zuntini , et al. 2020. “Tackling Rapid Radiations With Targeted Sequencing.” Frontiers in Plant Science 10: 1655. 31998342 10.3389/fpls.2019.01655 PMC6962237

[men70173-bib-0065] Larson, D. A. , M. W. Itgen , R. D. Denton , and M. W. Hahn . 2026. “Reconsidering Cytonuclear Discordance in the Genomic Age.” EcoEvoRxiv 80, no. 1: 1–14. 10.1093/evolut/qpaf201. 41055410

[men70173-bib-0066] Lehtonen, J. , and R. Lanfear . 2014. “Generation Time, Life History and the Substitution Rate of Neutral Mutations.” Biology Letters 10, no. 11: 20140801. 25428931 10.1098/rsbl.2014.0801 PMC4261869

[men70173-bib-0067] Li, H. , and R. Durbin . 2009. “Fast and Accurate Short Read Alignment With Burrows‐Wheeler Transform.” Bioinformatics 25, no. 14: 1754–1760. 19451168 10.1093/bioinformatics/btp324 PMC2705234

[men70173-bib-0068] Li, H. T. , Y. Luo , L. Gan , et al. 2021. “Plastid Phylogenomic Insights Into Relationships of All Flowering Plant Families.” BMC Biology 19: 1–13. 34711223 10.1186/s12915-021-01166-2 PMC8555322

[men70173-bib-0069] Li, Z. Z. , S. Lehtonen , and J. M. Chen . 2023. “The Dynamic History of Plastome Structure Across Aquatic Subclass Alismatidae.” BMC Plant Biology 23: 125. 36869282 10.1186/s12870-023-04125-x PMC9985265

[men70173-bib-0070] Liu, Y. , M. G. Johnson , C. J. Cox , et al. 2019. “Resolution of the Ordinal Phylogeny of Mosses Using Targeted Exons From Organellar and Nuclear Genomes.” Nature Communications 10: 1485. 10.1038/s41467-019-09454-w PMC6445109 30940807

[men70173-bib-0071] Mai, U. , and S. Mirarab . 2018. “TreeShrink: Fast and Accurate Detection of Outlier Long Branches in Collections of Phylogenetic Trees.” BMC Genomics 19, no. 5: 23–40. 29745847 10.1186/s12864-018-4620-2 PMC5998883

[men70173-bib-0072] Marinho, L. C. , L. Cai , X. Duan , et al. 2019. “Plastomes Resolve Generic Limits Within Tribe Clusieae (Clusiaceae) and Reveal the New Genus *Arawakia* .” Molecular Phylogenetics and Evolution 134: 142–151. 30743062 10.1016/j.ympev.2019.02.005

[men70173-bib-0073] McKain, M. R. , M. G. Johnson , S. Uribe‐Convers , D. Eaton , and Y. Yang . 2018. “Practical Considerations for Plant Phylogenomics.” Applications in Plant Sciences 6, no. 3: E1038. 29732268 10.1002/aps3.1038 PMC5895195

[men70173-bib-0074] McLay, T. G. , J. L. Birch , B. F. Gunn , et al. 2021. “New Targets Acquired: Improving Locus Recovery From the Angiosperms353 Probe Set.” Applications in Plant Sciences 9, no. 7: E11420. 10.1002/aps3.11420 PMC8312740 34336399

[men70173-bib-0075] Meseguer, A. S. , J. J. Aldasoro , and I. Sanmartín . 2013. “Bayesian Inference of Phylogeny, Morphology and Range Evolution Reveals a Complex Evolutionary History in St. John's Wort (*Hypericum*).” Molecular Phylogenetics and Evolution 67, no. 2: 379–403. 23435266 10.1016/j.ympev.2013.02.007

[men70173-bib-0076] Meseguer, A. S. , J. M. Lobo , J. Cornuault , et al. 2018. “Reconstructing Deep‐Time Palaeoclimate Legacies in the Clusioid Malpighiales Unveils Their Role in the Evolution and Extinction of the Boreotropical Flora.” Global Ecology and Biogeography 27, no. 5: 616–628.

[men70173-bib-0077] Meseguer, A. S. , J. M. Lobo , R. Ree , D. J. Beerling , and I. Sanmartín . 2015. “Integrating Fossils, Phylogenies, and Niche Models Into Biogeography to Reveal Ancient Evolutionary History: The Case of *Hypericum* (Hypericaceae).” Systematic Biology 64, no. 2: 215–232. 25398444 10.1093/sysbio/syu088 PMC4380036

[men70173-bib-0078] Meyer, M. , and M. Kircher . 2010. “Illumina Sequencing Library Preparation for Highly Multiplexed Target Capture and Sequencing.” Cold Spring Harbor Protocols 2010, no. 6: Pdb‐Prot5448. 10.1101/pdb.prot5448 20516186

[men70173-bib-0079] Minh, B. Q. , H. A. Schmidt , O. Chernomor , et al. 2020. “IQ‐TREE 2: New Models and Efficient Methods for Phylogenetic Inference in the Genomic Era.” Molecular Biology and Evolution 37, no. 5: 1530–1534. 32011700 10.1093/molbev/msaa015 PMC7182206

[men70173-bib-0080] Mirarab, S. 2019. “Species Tree Estimation Using ASTRAL: Practical Considerations.” *arXiv*. 1904.03826.

[men70173-bib-0081] Mitchell, N. , P. O. Lewis , E. M. Lemmon , A. R. Lemmon , and K. E. Holsinger . 2017. “Anchored Phylogenomics Improves the Resolution of Evolutionary Relationships in the Rapid Radiation of *Protea* L.” American Journal of Botany 104, no. 1: 102–115. 28104589 10.3732/ajb.1600227

[men70173-bib-0082] Moore‐Pollard, E. R. , D. S. Jones , and J. R. Mandel . 2024. “Compositae‐Paraloss‐1272: A Complementary Sunflower‐Specific Probe Set Reduces Paralogs in Phylogenomic Analyses of Complex Systems.” Applications in Plant Sciences 12, no. 1: E11568. 38369976 10.1002/aps3.11568 PMC10873820

[men70173-bib-0083] Musker, S. D. , N. M. Nürk , and M. D. Pirie . 2025. “Maximising Informativeness for Target Capture‐Based Phylogenomics in *Erica* (Ericaceae).” PhytoKeys 251: 87–118. 39867481 10.3897/phytokeys.251.136373 PMC11758362

[men70173-bib-0084] Nge, F. J. , E. Biffin , K. R. Thiele , and M. Waycott . 2021. “Reticulate Evolution, Ancient Chloroplast Haplotypes, and Rapid Radiation of the Australian Plant Genus *Adenanthos* (Proteaceae).” Frontiers in Ecology and Evolution 8: 616741.

[men70173-bib-0085] Notis, C. 2004. Phylogeny and Character Evolution of Kielmeyeroideae (Clusiaceae) Based on Molecular and Morphological Data (Doctoral Dissertation). University of Florida.

[men70173-bib-0086] Nürk, N. M. , S. Madriñán , M. A. Carine , M. W. Chase , and F. R. Blattner . 2013. “Molecular Phylogenetics and Morphological Evolution of St. John's Wort (*Hypericum*; Hypericaceae).” Molecular Phylogenetics and Evolution 66, no. 1: 1–16. 23058779 10.1016/j.ympev.2012.08.022

[men70173-bib-0087] Nürk, N. M. , S. Uribe‐Convers , B. Gehrke , D. C. Tank , and F. R. Blattner . 2015. “Oligocene Niche Shift, Miocene Diversification‐Cold Tolerance and Accelerated Speciation Rates in the St. John's Worts (*Hypericum*, Hypericaceae).” BMC Evolutionary Biology 15: 1–13. 25944090 10.1186/s12862-015-0359-4 PMC4422466

[men70173-bib-0088] Ogutcen, E. , C. Christe , K. Nishii , N. Salamin , M. Möller , and M. Perret . 2021. “Phylogenomics of Gesneriaceae Using Targeted Capture of Nuclear Genes.” Molecular Phylogenetics and Evolution 157: 107068. 33422648 10.1016/j.ympev.2021.107068

[men70173-bib-0089] Paradis, E. , and K. Schliep . 2019. “ *ape* 5.0: ‘An Environment for Modern Phylogenetics and Evolutionary Analyses in R’.” Bioinformatics 35, no. 3: 526–528. 30016406 10.1093/bioinformatics/bty633

[men70173-bib-0090] Peredo, E. L. , U. M. King , and D. H. Les . 2013. “The Plastid Genome of *Najas flexilis* : Adaptation to Submersed Environments Is Accompanied by the Complete Loss of the NDH Complex in an Aquatic Angiosperm.” PLoS One 8, no. 7: E68591. 23861923 10.1371/journal.pone.0068591 PMC3701688

[men70173-bib-0091] Philbrick, C. T. , and A. R. Novelo . 1995. “New World Podostemaceae: Ecological and Evolutionary Enigmas.” Brittonia 47: 210–222.

[men70173-bib-0092] Philbrick, C. T. , B. R. Ruhfel , and C. P. Bove . 2018. “Contributions to the Taxonomy of *Rhyncholacis* (Podostemaceae): Evidence of Monophyly, Description of a New Species, and Transfer of the Monotypic *Macarenia* .” Phytotaxa 357, no. 2: 107–116.

[men70173-bib-0093] Price, M. N. , P. S. Dehal , and A. P. Arkin . 2010. “FastTree 2—Approximately Maximum‐Likelihood Trees for Large Alignments.” PLoS One 5, no. 3: E9490. 20224823 10.1371/journal.pone.0009490 PMC2835736

[men70173-bib-0094] Qiu, S. , K. Xia , Y. Yang , Q. Wu , and Z. Zhao . 2023. “Mechanisms Underlying the C3–CAM Photosynthetic Shift in Facultative CAM Plants.” Horticulturae 9, no. 3: 398.

[men70173-bib-0095] R Core Team . 2024. R: A Language and Environment for Statistical Computing. R Foundation for Statistical Computing. https://www.R‐project.org/.

[men70173-bib-0096] Rambaut, A. 2018. FigTree Version 1.4.4. Institute of Evolutionary Biology, University of Edinburgh. https://github.com/rambaut/figtree.

[men70173-bib-0097] Ramírez‐Barahona, S. , H. Sauquet , and S. Magallón . 2020. “The Delayed and Geographically Heterogeneous Diversification of Flowering Plant Families.” Nature Ecology & Evolution 4, no. 9: 1232–1238. 32632260 10.1038/s41559-020-1241-3

[men70173-bib-0098] Robson, N. K. B. 1977. “Studies in the Genus *Hypericum* L. (Guttiferae). I. Infrageneric Classification.” Bulletin of the Natural History Museum. Botany Series 5: 295–355.

[men70173-bib-0099] Ruhfel, B. R. , V. Bittrich , C. P. Bove , et al. 2011. “Phylogeny of the Clusioid Clade (Malpighiales): Evidence From the Plastid and Mitochondrial Genomes.” American Journal of Botany 98, no. 2: 306–325. 21613119 10.3732/ajb.1000354

[men70173-bib-0100] Ruhfel, B. R. , C. P. Bove , C. T. Philbrick , and C. C. Davis . 2016. “Dispersal Largely Explains the Gondwanan Distribution of the Ancient Tropical Clusioid Plant Clade.” American Journal of Botany 103, no. 6: 1117–1128. 27335391 10.3732/ajb.1500537

[men70173-bib-0101] Sanderson, B. J. , S. P. Difazio , Q. C. Cronk , T. Ma , and M. S. Olson . 2020. “A Targeted Sequence Capture Array for Phylogenetics and Population Genomics in the Salicaceae.” Applications in Plant Sciences 8, no. 10: E11394. 33163293 10.1002/aps3.11394 PMC7598885

[men70173-bib-0102] Schneider, J. V. , T. Jungcurt , D. Cardoso , et al. 2021. “Phylogenomics of the Tropical Plant Family Ochnaceae Using Targeted Enrichment of Nuclear Genes and 250+ Taxa.” Taxon 70, no. 1: 48–71.

[men70173-bib-0103] Shah, T. , F. H. Mashimba , H. O. Suleiman , et al. 2022. “Phylogenetics of *Ochna* (Ochnaceae) and a New Infrageneric Classification.” Botanical Journal of the Linnean Society 198, no. 4: 361–381.

[men70173-bib-0104] Shee, Z. Q. , D. G. Frodin , R. Cámara‐Leret , and L. Pokorny . 2020. “Reconstructing the Complex Evolutionary History of the Papuasian *Schefflera* Radiation Through Herbariomics.” Frontiers in Plant Science 11: 258. 32265950 10.3389/fpls.2020.00258 PMC7099051

[men70173-bib-0105] Simmons, M. P. , and J. Gatesy . 2021. “Collapsing Dubiously Resolved Gene‐Tree Branches in Phylogenomic Coalescent Analyses.” Molecular Phylogenetics and Evolution 158: 107092. 33545272 10.1016/j.ympev.2021.107092

[men70173-bib-0106] Siniscalchi, C. M. , O. Hidalgo , L. Palazzesi , et al. 2021. “Lineage‐Specific vs. Universal: A Comparison of the Compositae1061 and Angiosperms353 Enrichment Panels in the Sunflower Family.” Applications in Plant Sciences 9, no. 7: E11422. 10.1002/aps3.11422 PMC8312747 34336403

[men70173-bib-0107] Slimp, M. , L. D. Williams , H. Hale , and M. G. Johnson . 2021. “On the Potential of Angiosperms353 for Population Genomic Studies.” Applications in Plant Sciences 9, no. 7: 11419. 10.1002/aps3.11419 PMC8312745 34336401

[men70173-bib-0108] Smith, S. A. , and M. J. Donoghue . 2008. “Rates of Molecular Evolution Are Linked to Life History in Flowering Plants.” Science 322, no. 5898: 86–89. 18832643 10.1126/science.1163197

[men70173-bib-0109] Soltis, P. S. , R. A. Folk , and D. E. Soltis . 2019. “Darwin Review: Angiosperm Phylogeny and Evolutionary Radiations.” Proceedings of the Royal Society B: Biological Sciences 286, no. 1899: 20190099.

[men70173-bib-0110] Soto Gomez, M. , L. Pokorny , M. B. Kantar , et al. 2019. “A Customized Nuclear Target Enrichment Approach for Developing a Phylogenomic Baseline for *Dioscorea* Yams (Dioscoreaceae).” Applications in Plant Sciences 7, no. 6: e11254. 31236313 10.1002/aps3.11254 PMC6580989

[men70173-bib-0111] Steenwyk, J. L. , Y. Li , X. Zhou , X. X. Shen , and A. Rokas . 2023. “Incongruence in the Phylogenomics Era.” Nature Reviews Genetics 24, no. 12: 834–850. 10.1038/s41576-023-00620-x PMC11499941 37369847

[men70173-bib-0112] Stevens, P. F. 2007. “Clusiaceae‐Guttiferae.” In Flowering Plants. Eudicots. The Families and Genera of Vascular Plants, Volume 9, edited by K. Kubitzki , 48–66. Springer.

[men70173-bib-0113] Stull, G. W. , K. K. Pham , P. S. Soltis , and D. E. Soltis . 2023. “Deep Reticulation: The Long Legacy of Hybridization in Vascular Plant Evolution.” Plant Journal 114, no. 4: 743–766. 10.1111/tpj.16142 36775995

[men70173-bib-0114] Stull, G. W. , P. S. Soltis , D. E. Soltis , M. A. Gitzendanner , and S. A. Smith . 2020. “Nuclear Phylogenomic Analyses of Asterids Conflict With Plastome Trees and Support Novel Relationships Among Major Lineages.” American Journal of Botany 107, no. 5: 790–805. 32406108 10.1002/ajb2.1468

[men70173-bib-0115] Sun, M. , R. Naeem , J. X. Su , et al. 2016. “Phylogeny of the Rosidae: A Dense Taxon Sampling Analysis.” Journal of Systematics and Evolution 54, no. 4: 363–391.

[men70173-bib-0116] Takhtajan, A. 1997. Diversity and Classification of Flowering Plants. Columbia University Press.

[men70173-bib-0117] Thomas, S. K. , X. Liu , Z. Y. Du , et al. 2021. “Comprehending Cornales: Phylogenetic Reconstruction of the Order Using the Angiosperms353 Probe Set.” American Journal of Botany 108, no. 7: 1112–1121. 34263456 10.1002/ajb2.1696 PMC8361741

[men70173-bib-0118] Tippery, N. P. , C. T. Philbrick , C. P. Bove , and D. H. Les . 2011. “Systematics and Phylogeny of Neotropical Riverweeds (Podostemaceae: Podostemoideae).” Systematic Botany 36, no. 1: 105–111.

[men70173-bib-0119] Trad, R. J. , F. N. Cabral , V. Bittrich , S. R. D. Silva , and M. C. E. Do Amaral . 2021. “Calophyllaceae Plastomes, Their Structure and Insights in Relationships Within the Clusioids.” Scientific Reports 11, no. 1: 20712. 34671062 10.1038/s41598-021-99178-z PMC8528878

[men70173-bib-0120] Ufimov, R. , V. Zeisek , S. Píšová , et al. 2021. “Relative Performance of Customized and Universal Probe Sets in Target Enrichment: A Case Study in Subtribe Malinae.” Applications in Plant Sciences 9, no. 7: E11442. 34336405 10.1002/aps3.11442 PMC8312748

[men70173-bib-0121] Van Bel, M. , S. Proost , E. Wischnitzki , et al. 2012. “Dissecting Plant Genomes With the PLAZA Comparative Genomics Platform.” Plant Physiology 158, no. 2: 590–600. 22198273 10.1104/pp.111.189514 PMC3271752

[men70173-bib-0122] Villaverde, T. , I. Larridon , T. Shah , et al. 2023. “Phylogenomics Sheds New Light on the Drivers Behind a Long‐Lasting Systematic Riddle: The Figwort Family Scrophulariaceae.” New Phytologist 240, no. 4: 1601–1615. 36869601 10.1111/nph.18845

[men70173-bib-0123] Villaverde, T. , L. Pokorny , S. Olsson , et al. 2018. “Bridging the Micro‐ and Macroevolutionary Levels in Phylogenomics: Hyb‐Seq Solves Relationships From Populations to Species and Above.” New Phytologist 220, no. 2: 636–650. 30016546 10.1111/nph.15312

[men70173-bib-0124] Wawra, A. 1886. “Ternstroemiaceae.” In Flora Brasiliensis, Volume 12, edited by C. F. P. Martius , A. G. Eichler , and I. Urban , 258–334. Frid. Fleischer.

[men70173-bib-0125] Waycott, M. , K. J. Van Dijk , and E. Biffin . 2021. “A Hybrid Capture RNA Bait Set for Resolving Genetic and Evolutionary Relationships in Angiosperms From Deep Phylogeny to Intraspecific Lineage Hybridization.” *bioRxiv*: 2021.09.06.456727.

[men70173-bib-0126] Weitemier, K. , S. C. Straub , R. C. Cronn , et al. 2014. “Hyb‐Seq: Combining Target Enrichment and Genome Skimming for Plant Phylogenomics.” Applications in Plant Sciences 2, no. 9: 1400042. 10.3732/apps.1400042 PMC4162667 25225629

[men70173-bib-0127] Wen, D. , Y. Yu , J. Zhu , and L. Nakhleh . 2018. “Inferring Phylogenetic Networks Using PhyloNet.” Systematic Biology 67, no. 4: 735–740. 29514307 10.1093/sysbio/syy015 PMC6005058

[men70173-bib-0128] WFO . 2024. “World Flora Online.” Accessed 24 January 2024. Worldfloraonline.org.

[men70173-bib-0129] Wurdack, K. J. , and C. C. Davis . 2009. “Malpighiales Phylogenetics: Gaining Ground on One of the Most Recalcitrant Clades in the Angiosperm Tree of Life.” American Journal of Botany 96, no. 8: 1551–1570. 21628300 10.3732/ajb.0800207

[men70173-bib-0130] Xi, Z. , B. R. Ruhfel , H. Schaefer , et al. 2012. “Phylogenomics and a Posteriori Data Partitioning Resolve the Cretaceous Angiosperm Radiation Malpighiales.” Proceedings of the National Academy of Sciences of the United States of America 109, no. 43: 17519–17524. 23045684 10.1073/pnas.1205818109 PMC3491498

[men70173-bib-0131] Yardeni, G. , J. Viruel , M. Paris , et al. 2022. “Taxon‐Specific or Universal? Using Target Capture to Study the Evolutionary History of Rapid Radiations.” Molecular Ecology Resources 22, no. 3: 927–945. 34606683 10.1111/1755-0998.13523 PMC9292372

[men70173-bib-0132] Zhang, C. , M. Rabiee , E. Sayyari , and S. Mirarab . 2018. “ASTRAL‐III: Polynomial Time Species Tree Reconstruction From Partially Resolved Gene Trees.” BMC Bioinformatics 19: 15–30. 29745866 10.1186/s12859-018-2129-y PMC5998893

[men70173-bib-0133] Zuntini, A. R. , T. Carruthers , O. Maurin , et al. 2024. “Phylogenomics and the Rise of the Angiosperms.” Nature 629: 843–850. 38658746 10.1038/s41586-024-07324-0 PMC11111409

